# Synergistic Optimization Between Chromium Local Coordination States Toward Self‐Powered High‐Repeatability Near‐Infrared Mechanoluminescence

**DOI:** 10.1002/advs.202518364

**Published:** 2025-11-07

**Authors:** Yao Xiao, Puxian Xiong, Gaochao Liu, Yongsheng Sun, Xuesong Wang, Pan Zheng, Enhai Song, Jiulin Gan

**Affiliations:** ^1^ State Key Laboratory of Luminescent Materials and Devices Institute of Optical Communication Materials Guangdong Engineering Technology Research and Development Center of Special Optical Fiber Materials and Devices Guangdong Provincial Key Laboratory of Fiber Laser Materials and Applied Techniques South China University of Technology Guangzhou 510640 China; ^2^ Department of Electrical and Electronic Engineering The University of Hong Kong Hong Kong 999077 China

**Keywords:** high repeatability, mechanoluminescence, multimode sensing, Cr^3+^, near‐infrared, self‐powering

## Abstract

Self‐powered mechanoluminescence (S‐ML) elastomer with near‐infrared (NIR) emission exhibits great potential for the next generation of bio‐imaging, bio‐sensing, and human‐machine interaction fields. However, due to the lack of understanding of the mechanical‐photon conversion mechanism, the emission efficiency and cycling stability of ML materials reported so far are still unable to meet the application needs. Herein, high‐repeatability (>10000 times) and tunable near‐infrared (650–1000 nm) mechanoluminescent materials are reported by optimizing Cr^3+^ local coordination states in a simple centrosymmetric MgO host. The constructed electron transfer model among multiple Cr^3+^ ion states (isolated Cr^3+^, Cr^3+^ pair, and Cr^3+^ cluster) reveals the structure‐activity relationship between local piezoelectricity and photoelectric output. Theoretical calculations and experimental results reveal that the heterovalent substitution of Cr^3+^ ions promotes the [MgO_6_] distortion to activate the nearest neighboring defect to form suitable intermediate gap states, facilitating stress‐driven electron tunneling to Cr^3+^ states. Proof‐of‐concept multi‐layered bright field sensing and imaging is developed with all‐round interactive NIR tactile perception. This work not only provides a high‐repeatability NIR ML phosphor but also establishes the integrated thinking mode for material‐performance‐device rational design.

## Graphical abstract

Herein, a Cr^3+^‐activated tunable near‐infrared (650–1000 nm) self‐powered mechanoluminescence material with high intensity and repeatability is innovatively developed. This work focuses on optimizing multiple Cr^3+^ local coordination environments (isolated Cr^3+^, Cr^3+^ pair, and Cr^3+^ cluster) to deeply understand the electron transfer mechanism behind the self‐powered mechanoluminescence, and proposes a proof‐of‐concept application model in the field of NIR bio‐imaging and sensing, showing extraordinary potential for intelligent sensors, wearable technology, and biomedical applications.

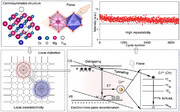

## 1 Introduction

Mechanoluminescent (ML) materials have emerged as a pivotal class of functional media for mechanical sensing and imaging due to the unique mechanical‐light conversion characteristics. Compared with conventional photoluminescence or electroluminescence, ML can realize efficient conversion of mechanical energy in our daily life, achieving the renewability of energy. Leveraging real‐time, in situ optical visualization response, ML have broad application prospects in flexible display, smart sensing,^[^ ^1^ ^]^ artificial skin, bio‐medical/imaging^[^ ^2^, ^3^ ^]^ and other fields.^[^ ^4^ ^]^ Since two typical high‐performance ML materials (SrAl_2_O_4_: Eu^2+^ and ZnS: Mn^2+^) were discovered by Prof. Xu and co‐workers,^[^ ^5^ ^]^ ML spectral window has spanned from near‐ultraviolet (NUV) to near‐infrared (NIR) range. Contemporary ML compounds are classified into self‐powered (non‐pre‐irradiation: S‐ML) and trap‐controllable (pre‐irradiation: ML) types,^[^ ^6^, ^7^ ^]^ where the S‐powered characteristic allows ML materials to achieve ultra‐fast photoelectric response.^[^ ^8^ ^]^ Despite the rapid expansion of S‐ML phosphors, there are few with high performance and repeatability, especially in the NIR band. Among the limited S‐ML systems reported to date, only limited research, such as reported in ZnS: Mn and CaBa4(PO_4_)_3_Cl: Eu/PDMS, with exceptional mechanical durability (> 10 000 times) under continuous stretching conditions.^[^ ^9^, ^10^ ^]^ Owing to the excellent anti‐interference, biological tissue penetration, and spatial resolution capabilities in the NIR band, NIR S‐ML materials with high repeatability bring more possibilities for a wide range of applications in the ML field.^[^ ^11^, ^12^ ^]^

Although a handful of NIR S‐ML materials have been reported,^[^ ^13^, ^14^ ^]^ they universally exhibit inadequate repeatability and mechanical durability. Consequently, the urgent imperative is to engineer next‐generation NIR S‐ML phosphors whose performance satisfies the demands of advanced optoelectronics, but it is still a challenge. Limitations in the force‐to‐photoelectric conversion mechanisms of conventional NIR S‐ML material have hindered this development. Most researchers attribute the electron transfer mechanism of S‐ML to the contact‐separation model between the phosphor and the flexible substrate, while ignoring the properties of the phosphor itself.^[^ ^15^ ^]^ This will lead to a lack of sufficient understanding of key dynamic processes and coupling mechanisms, such as carrier generation, relaxation, and recombination, hindering the development. It would be of great significance in ML development if a universal physical model could be established and a novel S‐ML material could be developed to overcome the current bottlenecks in NIR repeatability and cyclic stability. In this regard, finding a suitable combination of host and emitter is the first step. Compared with lanthanide ions with narrow‐band *f*‐*f* transition,^[^ ^16^ ^]^ Cr^3+^‐activated inorganic oxide materials have received much attention due to tunable band emission, multiple Cr^3+^ ion coordination states, and high quantum efficiency.^[^ ^17^, ^18^ ^]^ Frankly, this is not an easy task considering the complex interactions between multiple Cr^3+^ ion coordination states, defect energy levels, and host structure.^[^ ^19^ ^]^ Following this point, Cr^3+^‐activated NIR S‐ML was developed in 2021^[^ ^20^ ^]^ and found in some compounds.^[^ ^21^ ^]^ Unlike the traditional non‐centrosymmetric piezoelectric structures, centrosymmetric compounds exhibit excellent photoelectrical signal decoupling ability owing to their excellent triboelectric and local piezoelectric effects.^[^ ^22^ ^]^ Meanwhile, the selection of a relatively simple host structure with few constituent elements is beneficial to exclude the influence of intrinsic defects in the host.

Previously, we confirmed that constructing heter‐junction in MgO/MgF_2_ system can greatly enhance its ML intensity,^[^ ^23^ ^]^ and self‐reduction method can also regulate ML performance in some ML host.^[^ ^24^ ^]^ In this work, we reported a NIR S‐ML phosphor with in situ and rapid photoelectric response by optimizing Cr^3+^ ion local coordination states in centrosymmetric MgO host. Notably, the S‐ML is stable and reproducible over 10000 times under continuous mechanical stimuli. Multiple magnesium vacancies (V_Mg_) and luminescent centers (isolated Cr^3+^, Cr^3+^ pair, and Cr^3+^ cluster) are confirmed, accounting for local piezoelectricity and triboelectricity during the emission process. The PL emission comes from isolated Cr^3+^ ion (^2^E_g_→^4^A_2g_), Cr^3+^ pair (^4^T_2g_→^4^A_2g_), and Cr^3+^ aggregation (Cr^3+^‐Cr^3+^→Cr^2+^‐Cr^4+^ intervalence charge transfer (IVCT). From density functional theory (DFT) calculations, Cr^3+^ doping promotes the deformation levels of [MgO_6_] and activates the nearest neighboring V_O_ and V_Mg_ defects to form additional defect states at the mid‐gap. These defect states are better matched to the Cr‐states to improve the electron transfer under mechanical stimuli via the tunneling process. The tilt of the energy band induced by the local piezoelectric field helps the trapped electrons to transfer to the Cr^3+^ ion, while the corresponding electrical signal is shown as piezo‐electric nanogenerators (PENG).^[^ ^25^ ^]^ Moreover, a multi‐layered tactile sensor is demonstrated to simulate the sensing and imaging mechanism to further explore the high‐end application NIR S‐ML material. The multimodal signals are integrated with artificial neural network (ANN) algorithms to achieve all‐round interactive tactile sensing and imaging, in which electrical signals are used to quantitatively evaluate the magnitude of the force, and optical signals provide 2D spatial mapping of the force, and then temperature changes are detected according to fluorescence intensity ratio (FIR) technology. In all, NIR S‐ML materials with the same high repeatability were discovered again after visible ZnS: Cu materials, and the corresponding S‐ML physical mechanism was proposed to guide the direction for the development of next‐generation ML materials.

## 2 Results and Discussion

### 2.1 Crystal Structure and Cr Local Environment Analysis

**Figure 1:**
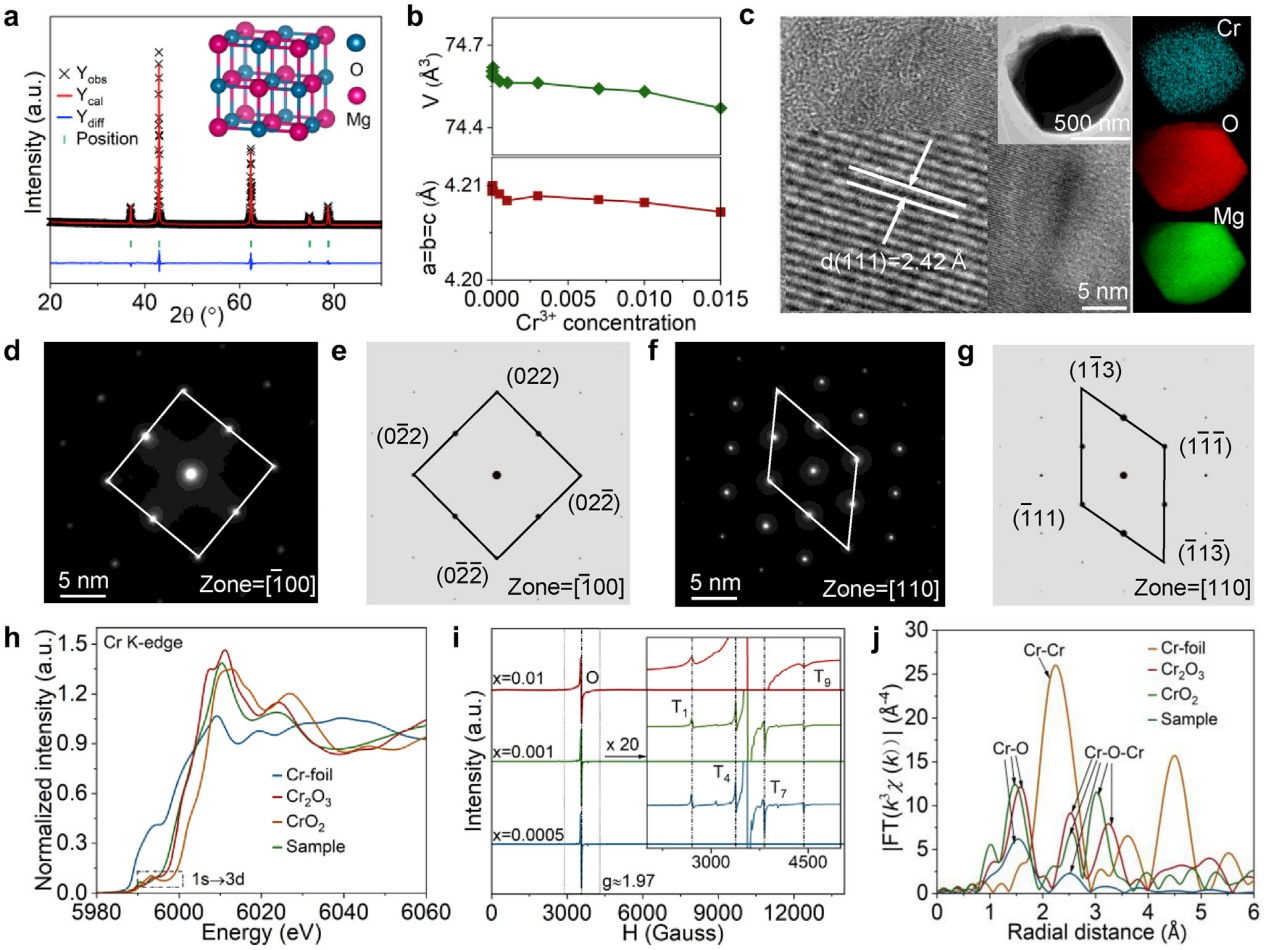
a) XRD Rietveld structure refinements of Mg_0.999_O: 0.001Cr^3+^. b) Cell parameters (*a*/*b*/*c*/*v*) of Rietveld refinement of Mg*_1‐x_* O: *x*Cr^3+^ (Fitting factor: *R_p_* = 8.67 %, *R_wp_* = 12.3 %, *χ^2^* = 5.11). c) HRTEM image displaying lattice fringes in d‐spacing. The white lines show the corresponding (hkl) planes, and the white arrows indicate the lattice spacing. The inset represents an HAADF‐STEM image and corresponding compositional analysis of an individual particle. d–g) FFT diffraction pattern taken from the square area of HR‐TEM image (MgO: Cr^3+^) and the simulated electron‐diffraction pattern (MgO) along the [‐100] zone axis and [110] zone axis. h) Cr K‐edge XANES data of Mg_0.995_O:0.005Cr^3+^, Cr‐foil, Cr_2_O_3_, and CrO_2_. i) EPR curve of Mg*_1‐x_* O: *x*Cr^3+^ and corresponding amplification curves between 2000 and 5000 G (T: Cr^3+^ in tetragonal coordination, O: Cr^3+^ in octahedral coordination). j) EXAFS and Fourier transform fitting curves in E‐space of the Mg_0.995_O: 0.005Cr^3+^, Cr‐foil, Cr_2_O_3_, and CrO_2_.

Pure phases MgO: *x*Cr^3+^ are confirmed first from XRD (Figure S1, Supporting Information). Subsequently, XRD Rietveld refinement confirms the cubic centrosymmetric structure (**Figure** **1**a; Figure S2, Supporting Information). The refined unit cell parameters (*a, b, c, V*) are changed due to radius mismatch between Cr^3+^ (0.615 Å, coordinated number (*CN*) = 6) and Mg^2+^ ions (0.72 Å, *CN* = 6), and the existence of multiple isolated Cr^3+^, Cr^3+^ pairs and Cr^3+^ clusters (Figure 1b; Table S1, Supporting Information). To maintain the charge balance, two Cr^3+^ can replace adjacent Mg^2+^ ions to form a Cr^3+^ pair and charged defects (Cr_Mg_ and V_Mg_ vacancy), where ICP‐AES confirms V_Mg_ existence (Figure S3, Supporting Information). Two types of V_Mg_ exist along the (001) and (110) crystal plane, leading to different local crystal environments to affecting Cr^3+^ ion emission^[^ ^26^ ^]^ (Figures S4–S6, Supporting Information). The local distortion is observed, which is conductive to enhancing Cr^3+^ absorption efficiency by breaking forbidden transitions^[^ ^27^ ^]^ (Equation S1 and Table S2, Supporting Information). The bond length between adjacent Cr^3+^ ion is less than 5 Å, facilitating Cr^3+^ pairs’ formation with defect assistance, as that in Mn^2+^‐Mn^2+^ dimer.^[^ ^28^ ^]^

The high‐resolution transmission electron microscopy (HRTEM) images and selected area electron diffraction (SAED) patterns verify the single‐crystal nature of MgO crystallization phase, and corresponding lattice fringe d‐spacings (2.42 Å) are slightly lower than that in pure MgO (2.43 Å) in (111) crystal plane (Figure 1c). The high‐angle annular dark‐field scanning transmission electron microscopy (HAADF‐STEM) further confirms the uniform distribution of Mg, O, and Cr within the particle (Figure 1c, insets). Meanwhile, the X‐ray Photoelectron Spectroscopy (XPS) survey curves of MgO: Cr^3+^ further demonstrate the presence of O, Cr, and Mg elements (Figure S7, Supporting Information). Corresponding fast Fourier transform (FFT) patterns indicate local crystal structure distortion in MgO due to hetero‐valent Cr^3+^ ion substitution (Figure 1d–g; Figure S8, Supporting Information). Because V_Mg_ defect is easier to form along (110) plane, the distortion degree in this crystal plane becomes larger, which is closely related to the generation of S‐ML and self‐powered PENG.

The K‐edge X‐ray absorption near‐edge structure (XANES) spectrum of Cr within MgO: 0.005Cr^3+^ is shown in Figure 1h, which determines the valence state and types of Cr. Compared to the higher binding energy of CrO_2_, the absorption energy of MgO: Cr^3+^ is located near Cr_2_O_3_, indicating that Cr is mainly present in +3. The corresponding first‐order derivative data also demonstrate this result, where the sample peak (red arrow) has a small difference with the standard Cr_2_O_3_ (Figure S9, Supporting Information). Moreover, high‐resolution XPS Cr 2*p* curves are consistent with Cr_2_O_3_, indicating that Cr^3+^ ion mainly exists in MgO (Figure S10a, Supporting Information). The pre‐edge peak (≈ 5990 eV) of Cr^3+^ ion displays a lower absorption coefficient due to the quadrupole‐allowed 1s→3d transition in octahedral sites. Meanwhile, no strong pre‐edge absorption is observed, revealing that Cr^4+^ is absent in the tetrahedral sites.^[^ ^29^ ^]^ Notably, to further research the local environment of Cr^3+^ ion, we performed electron paramagnetic resonance (EPR), EXAFS, and corresponding FFT analyses. Compared to the non‐kramers Cr^2+^ and Cr^4+^ ions with spins of *S* = 2 and *S* = 1, the transition metal Cr^3+^ (kramers, *S* = 3/2) ion has an apparent EPR signal at room temperature.^[^ ^30^ ^]^ EPR reveals that Cr^3+^ ions predominantly occupy octahedral sites,^[^ ^31^ ^]^ with a clear signal at high magnetic field range (*g* = 1.97) corresponding to Cr^3+^ pairs^[^ ^32^ ^]^ (Figure 1i). Because the oxygen vacancy (V_O_) signal is often present at this position^[^ ^33^ ^]^ (*g* = 2.003), the different rare earth and transition metal ions are doped in MgO host to confirm that the signal is independent of the V_O_ (Figure S10b, Supporting Information). EXAFS and corresponding FFT results further confirm the co‐existence of isolated Cr^3+^, Cr^3+^ pair, and Cr^3+^ cluster (Figure 1j; Figure S11 and Table S3, Supporting Information).

### 2.2 Multiple Cr^3+^ Photophysical Properties

**Figure 2:**
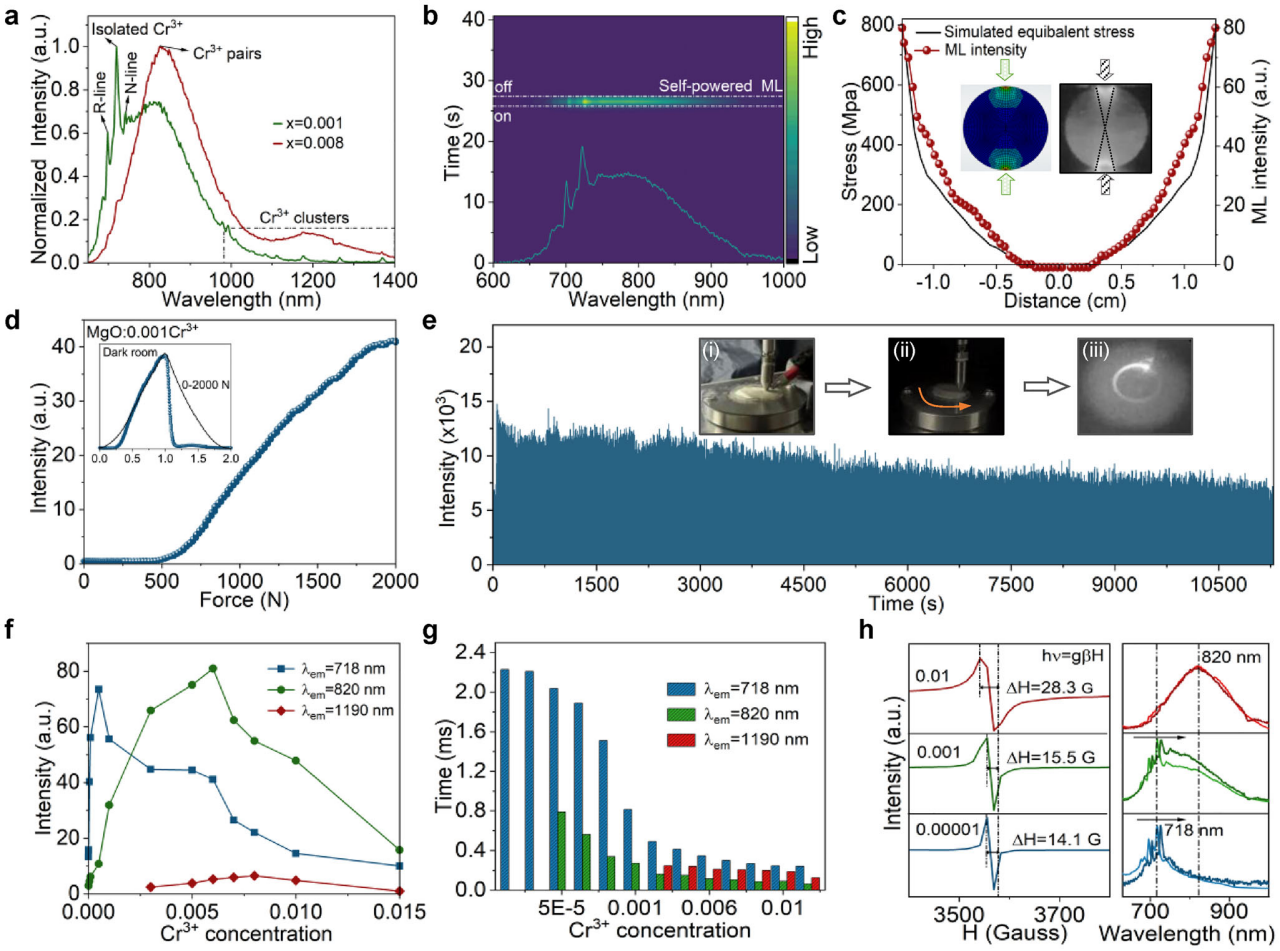
a) Normalized PL spectra of Mg*_1‐x_* O: *x*Cr^3+^ under 442 nm excitation. b) 2D color map surface graphs of S‐ML in Mg_0.999_O: 0.001Cr^3+^ with time decay under 2000 N loading. “On” and “Off” represent the start and end of force application, respectively. c) Comparison of experimental and simulated stress distribution of the Mg_0.999_O: 0.001Cr^3+^ pellet under 2000 N. Inset: finite element analysis 2D force image (left) and photograph (right) in the dark room during testing. d) Comparison of S‐ML intensity of Mg_0.999_O: 0.001Cr^3+^ pellet under applied pressure of 2000 N. Inset: comparison of triangular wave mode, where solid spheres and black lines represent the real‐time S‐ML intensities and applied pressure with time decay, respectively. e) Stability and repeatability tests 10 000 cycles under 5 N. Inset: schematic diagram of S‐ML stability and repeatability test. f) PL intensity of 718, 820, and 1190 nm for Mg*_1‐x_* O: *x*Cr^3+^ from 0.000005 to 0.015 under 442 nm light excitation. g) Fluorescence lifetime monitored at 718, 820, and 1190 nm of Mg*_1‐x_* O: *x*Cr^3+^ from 0.000005 to 0.015 under 442 nm excitation. h) EPR curves of Mg*_1‐x_* O: *x*Cr^3+^ (right), and corresponding ML (Dark lines) and PL (light lines) spectra (left).

Mg_0.999_O: 0.001Cr^3+^ displays broadband emission from isolated Cr^3+^ ion (^2^E_g_→^4^A_2g_) and Cr^3+^ pair (^4^T_2g_ → ^4^A_2g_) with near V_Mg_ defects^[^ ^34^ ^]^ **Figure**(2a). In addition, the long‐lifetime NIR‐II emission (peaked at 1190 nm) is attributed to the Cr^3+^ cluster under heavy Cr^3+^‐doped MgO (x ≥ 0.003), where IVCT accounts for this emission.^[^ ^35^ ^]^ Monitoring the corresponding PLE spectra, fluorescence lifetime, and diffuse reflection (DR) spectra also demonstrates that the continuous emission from NIR‐I to II is derived from Cr^3+^ ions (Figure S12, Supporting Information). The slightly red‐shifted PLE spectra indicate a variety of local coordination environments for Cr^3+^ ion, which is consistent with the previous structural analysis.^[^ ^36^ ^]^ The time‐resolved emission spectra (TRES) demonstrate the presence of multiple Cr^3+^ luminescence centers (Figure S13, Supporting Information). A similar S‐ML spectrum can be observed under 2000 N loading, indicating the same luminescent origin as PL (Figure 2b; Figure S14a, Supporting Information). The finite element analysis and 2D signal processing are used to further determine the mechano‐optical distribution information, which is conducive to optical‐based spatial force mapping analysis (Figure 2c). The ML signal distribution is matched to computational simulation results, where the force concentrates at the upper and lower ends of the hard pellet to produce high‐performance NIR S‐ML emission (Figure 2c, insets). Meanwhile, the significant S‐ML signal can also respond rapidly under various mechanical stimuli, promoting the multimodal tactile sensing in a variety of complex environments (Figure 2d; Figure S14b, Supporting Information). Notably, the S‐ML material also exhibited excellent photoelectric properties with stable and reproducible over 10000 times under continuous mechanical stimuli (Figure 2e; Figure S15, Supporting Information). The S‐ML intensity is weakened to 66.8% of its initial intensity after ten thousand cycles, which is attributed to damage to the material components during the long‐cycle test. The NIR‐II S‐ML is absent due to the low luminescence efficiency IVCT states and concentration quenching at high Cr^3+^ doping.

As Cr^3+^‐doped concentration increases, the PL intensity at 718 nm is gradually decreased, but 820 nm emission intensity changes differently due to concentration quenching and efficient energy transfer (ET) (Figure 2f). The ET process between multiple Cr^3+^ centers makes PL and ML spectra peak shift from 718 to 820 nm,^[^ ^37^ ^]^ where the ET pathways involve the electric dipole‐dipole (*d‐d*) and quadrupole‐quadrupole (*q‐q*) interactions (Figures S16 and S17 and Tables S4 and S5, Supporting Information). Fluorescence lifetime values are shortened at higher Cr^3+^ doping levels due to the super‐exchange interactions (Figure S18, Supporting Information).^[^ ^38^ ^]^ The IVCT transition occurs with a configurational transition between two neighboring Cr^3+^ ion, potentially with low absorption probability compared to the ^4^T_2_→^4^A_2_ inter‐configurational transition. Hence, the monitoring lifetime at 1190 nm is generally longer than 820 nm^[^ ^37^ ^]^ (Figure 2g). Compared to PL spectra, the slightly red‐shifted S‐ML spectra are observed, which is due to the fact that a stronger crystal field near Cr^3+^ ions may accelerate the level splitting of Cr^3+^ ion to generate a lower photon energy (Figure 2h, right). The optimal PL concentration is always higher than that of S‐ML, which is related with trap‐assisted long‐distance energy transfer between adjacent luminescent activators^[^ ^39^ ^]^ (Figure S19, Supporting Information). The broadening of the EPR peak corresponds to the increase of Cr^3+^‐doped concentration^[^ ^32^ ^]^ (Figure 2h, left). Moreover, the change trend of the EPR signal is similar to that in PL, which may be due to the increased Cr^3+^ clusters at high concentrations (Figure S20, Supporting Information).

### 2.3 Temperature‐Dependent Photophysical Mechanism

**Figure 3:**
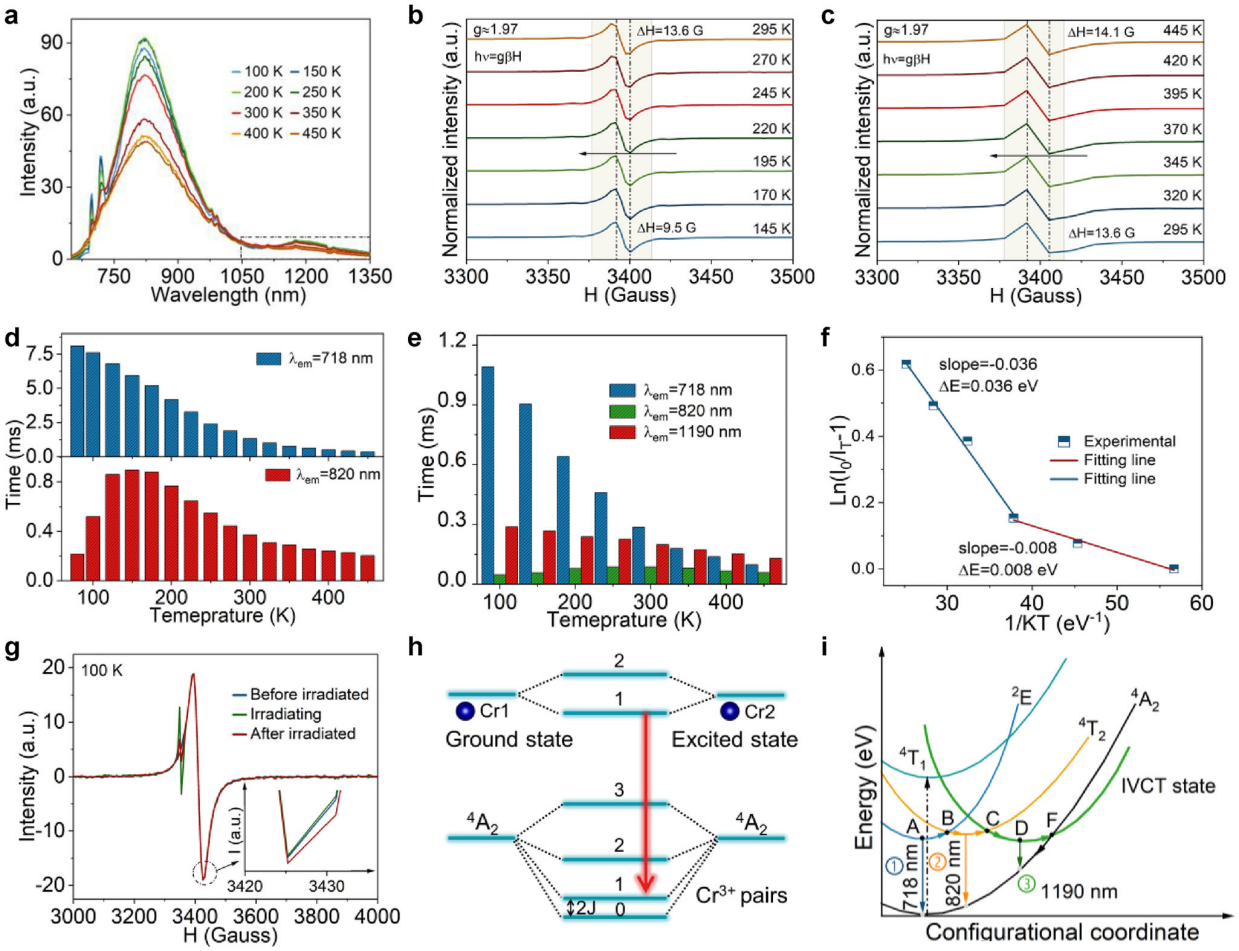
a) Temperature‐dependent PL spectra of Mg_0.992_O: 0.008Cr^3+^ from 100 to 450 K under 442 nm light excitation. b) Temperature‐dependent EPR curves from 145 to 295 K. c) Temperature‐dependent EPR curves from 295 to 445 K. d) Temperature‐dependent fluorescence lifetime of Mg_0.999_O: 0.001Cr^3+^ monitored at 718 and 820 nm under 442 nm excitation, respectively. e) Temperature‐dependent average fluorescence lifetime of Mg_0.992_O: 0.008Cr^3+^ monitored at 718, 820, and 1190 nm under 442 nm excitation, respectively. f) Decay fitted curve of Mg_0.992_O: 0.008Cr^3+^ for *Ln(I_0_/I_T_‐1)* versus temperature *1/KT*. The red line is low temperature, and the blue is high temperature. g) In situ EPR curves of Mg_0.992_O: 0.008Cr^3+^ with and without irradiation at 100 K. h) Energy level diagrams of Cr^3+^ pairs. i) Schematic configurational coordinate diagram with isolated Cr^3+^, Cr^3+^ pairs and Cr^3+^ clusters emission mechanism.

Temperature‐dependent PL and EPR in MgO: Cr^3+^ reveal the influence of Cr local environment and its photophysical mechanism mechanism on PL and S‐ML. As the temperature increases from 100 to 450 K, broadband PL is gradually emerging due to the thermal‐assisted occupation of the ^4^T_2g_ state **Figure** 3a. Meanwhile, the same phenomenon is observed in Mg_0.999_O: 0.001Cr^3+^ (Figure S21, Supporting Information). EPR curves are gradually widened from 9.5 to 14.1 G with increasing temperature, indicating ET process between Cr^3+^ pairs^[^ ^40^ ^]^ (Figure 3b,c). Notably, the NIR‐II emission (1190 nm) does not participate in the ET process, showing only PL intensity changes. The temperature‐dependent fluorescence lifetime for NIR‐I (718 and 820 nm) and NIR‐II (1190 nm) emission is plotted in Figure 3d,e and Figure S22 (Supporting Information). Clearly, the fluorescence lifetime at 718 nm is shortened with temperature, which is attributed to the increase in lattice vibration frequency, resulted phonon‐assisted non‐radiative relaxation process.^[^ ^27^ ^]^ There is a slight increase in the PL intensity and longer lifetime at 820 nm with temperature increasing, which is attributed to an accumulation of ^4^T_2_ excited‐state electrons (Figure 3d). Moreover, IVCT state formation below the ^4^T_2_ energy level prolongs electron residence toward longer lifetime (Figure 3e). The optimal doping content for 1190 nm is observed at x = 0.008, indicating two non‐radiative transition processes (Figure S23, Supporting Information). The activation energy of a nonradiative transition can be calculated by the Arrhenius formula, where *ΔE* represents the activation energy for the electron to move from the lowest IVCT excited state to the intersection with the ^4^T_2_ and ^4^A_2_ energy levels^[^ ^41^ ^]^ (Figure 3f; Equation S7, Supporting Information). As Cr^3+^ concentration increases, the electron transition from one Cr to another neighbor Cr ion becomes possible as in Cr^3+^‐Cr^3+^ → Cr^2+^‐Cr^4+^ process. Notably, IVCT is related to the partial oxidation and reduction process between Cr^3+^ clusters. However, the transfer of electrons occurs instantaneously, and it is difficult to monitor this process with conventional testing methods, so we conducted an in situ EPR experiment at 100 K and RT (Figure 3g; Figure S24, Supporting Information). Clearly, a novel EPR signal in the 3355 G range is observed during in situ irradiation, and the signal gradually disappears after stopping excitation. Meanwhile, Cr^3+^ pairs’ signal intensity is slightly decreased under irradiation, indicating that an electron can be released to generate the crystal lattice defect in the form of 2Cr^3+^ ± e− → Cr^4+^ and Cr^2+[^ ^35^ ^]^. The process of electron release and transition reveals the coexistence of additional luminescent centers regarding single Cr^2+^/Cr^4+^. Those EPR signals have a close relationship with the Cr^3+^ clusters/pairs interaction and non‐equivalence substitution defects. Notably, Cr^3+^ ions in a highly distorted octahedral environment can activate the lattice defects and electron transfer under irradiation, forming the hetero‐valent Cr^3+^‐Cr^3+^ → Cr^2+^‐Cr^4+^ pairs confirmed by in situ EPR results. Notably, Cr^3+^ pairs can also show additional energy level distribution to regulate S‐ML and PL emission owing to interionic interactions. The ground state of the Cr^3+^ pairs originates from the exchange coupling between the ground states of two individual Cr^3+^ ions (Cr1 and Cr2), where both ions remain in their respective ground state configurations (Figure 3h). In contrast, the first excited state of the pair emerges when Cr1 resides in its ground state while Cr2 transitions to an excited state, with these two distinct states being coupled via exchange interactions.^[^ ^42^ ^]^ Importantly, the excitation energies for both isolated Cr^3+^ ions and Cr^3+^ pairs are governed by the absorption characteristics of Cr2, resulting in identical spectral peak positions and line shapes in the PLE spectra from both isolated Cr^3+^ ions and Cr^3+^ pairs. Because the Cr^3+^ pairs are spin‐allowed transitions of *ΔS* = 0, the magnetic interaction of the Cr^3+^ pairs can change the spin configuration of electrons, further breaking the *d*‐*d* transition spin prohibition‐selective law of the isolated Cr^3+^ ion, and then leading to higher transition probabilities and contributing to broadband emission.^[^ ^43^ ^]^ More detailed analysis is shown in Equations S8 and S9 (Supporting Information).

Based on the above discussion results, the photophysical mechanism of Cr^3+^‐activated MgO can be explained by a configuration coordinate diagram (Figure 3i). First, the electrons are pumped from the ground state (^4^A_2_) to the higher excited state (^4^T_1_) and then undergo a non‐radiative transition from ^4^T_1_ to ^2^E state, followed by the radiative transition with narrow‐band (718 nm) and N/R‐line emission (Process 1). Second, the formation of Cr^3+^ pairs will shorten the distance between ^2^E and ^4^T_2_ energy levels, promoting the energy migration efficiency for isolated Cr^3+^ ions (Process A to B). This will lead to a red‐shift and broadband emission (820 nm), where the emission is derived from the radiative transition from ^4^T_2_ to ^4^A_2_ state (Process 2). The introduction of more Cr^3+^ ions will affect the crystal field splitting strength around the Cr^3+^ ions and change the emission energy.^[^ ^36^ ^]^ The electrons will be transferred from Cr^3+^ pairs to clusters, corresponding to the ^4^T_2_’s migration to the IVCT state (Process C to D), which results in the radiative transition with NIR‐II broad‐band emission (Process 3). The electron transition in the IVCT state can be divided into two processes: the oxidation of one Cr^3+^ ion (Cr^3+^ → e^−^ + Cr^4+^) and the reduction of another Cr^3+^ ion (Cr^3+^ + e^−^ → Cr^2+^), in which the position of the IVCT state is below the excited state ^4^T_2_ of Cr^3+^ ion. Notably, a similar transition process is widely found in phosphors doped with rare earth and Bi ions.^[^ ^44^ ^]^ Clearly, there is a strong energy transfer process from Cr^3+^ to Ni^2+^ ions, which is caused by the adaptation of the IVCT state formed by Cr^3+^ clusters to the energy level of Ni^2+[^ ^45^ ^]^ (Figure S25, Supporting Information). In all, the relationship between different Cr^3+^ ion coordination states and PL was successfully demonstrated, and the different luminescence region laid a good foundation for the MgO:Cr^3+^ as a good sensing material such as for temperature.^[^ ^46^ ^]^

### 2.4 Self‐Powered Photo‐Electric Response Mechanism

Notably, the existence of multiple Cr^3+^ ion coordination states determines the origin and performance of PL and S‐ML. It is necessary to understand and explore scientifically the causes of S‐ML to promote the development of high‐performance NIR ML materials. Compared to the other Cr^3+^ ion coordination states, Cr^3+^ pairs can produce significant S‐ML emission, where the signal of Cr^3+^ pairs can be observed by the EPR curves (*g* = 1.97) (Table S6, Supporting Information). The Cr^3+^ pairs are conducive to the S‐ML emission, while Cr^3+^ clusters prevent it, because Cr^3+^ clusters are formed at high Cr^3+^ doping, while the IVCT state can block the original electron transition between ^4^T_2_ and ^2^E under mechanical stimuli. The weak S‐ML under low‐concentration Cr^3+^ ion doping is attributed to the lack in ET process between Cr^3+^ ions.

**Figure 4:**
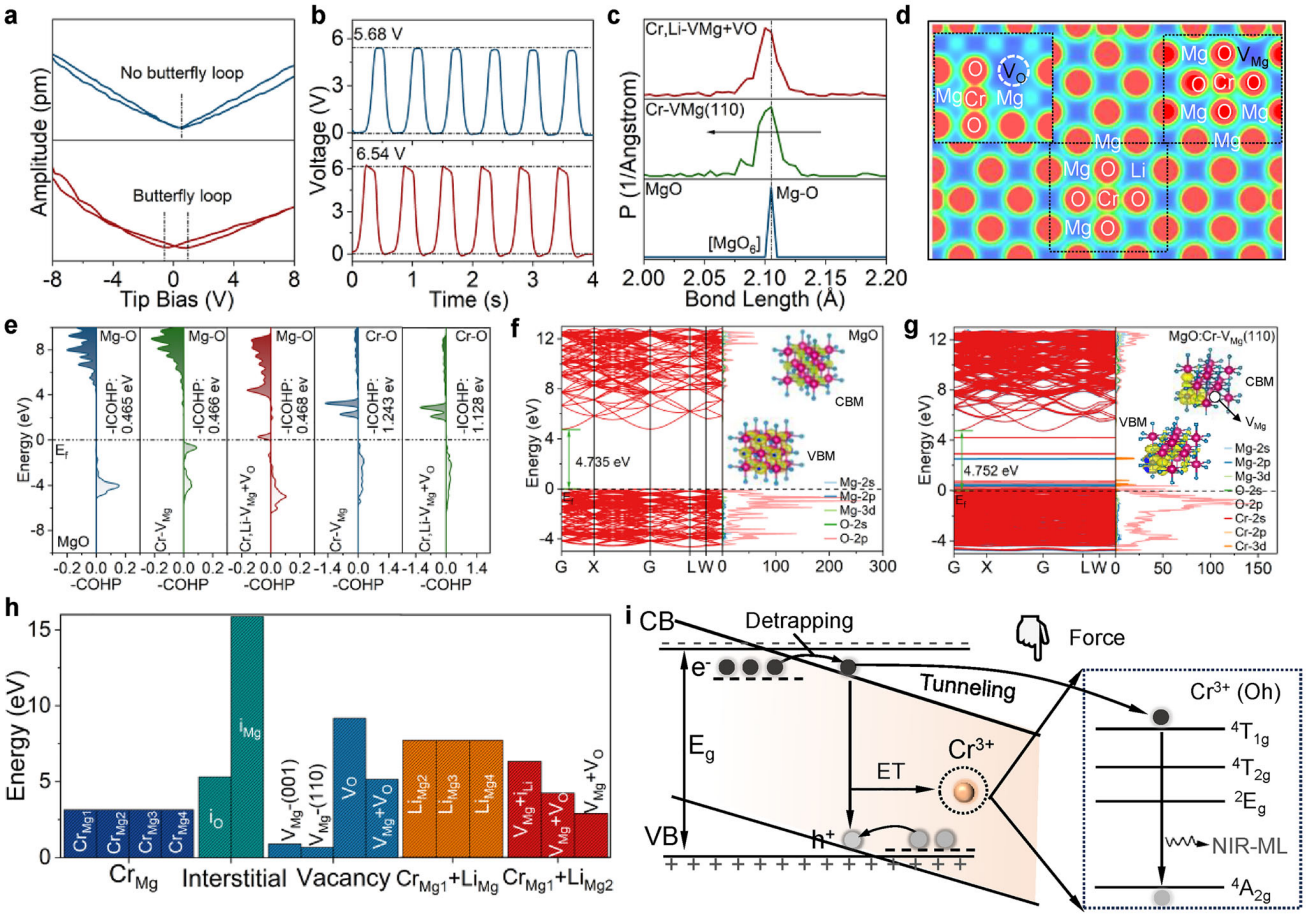
a) PFM amplitude and butterfly loops of Mg_0.999_O: 0.001Cr^3+^ (down) and MgO (up) under a tip bias in the range of ±8 V. b) The stable voltage output for Mg_0.999_O: 0.001Cr^3+^ (up) and MgO (down) under 5 N force. (c) The bond length distribution comparisons of Mg‐O in MgO, MgO: Cr^3+^ with V_Mg,_ and MgO: Li^+^, Cr^3+^ with V_Mg_ and Vo. d) The 2D slice of the charge distribution in MgO: Li^+^, Cr^3+^ with V_Mg_ and Vo defects upon (100) direction. e) Crystal orbital Hamilton population curves of Mg‐O/Cr‐O under different models. The Fermi energy is indicated as a dotted line. f) Band structure and electronic densities of state and the Fermi energy level for MgO. (g) Band structure and electronic densities of state and the Fermi energy level for MgO: Cr with. h) The formation energies of different types of defects. i) S‐ML mechanism schematic diagram.

The Cr^3+^ pairs are crucial for the generation of S‐ML, so we further regulate the coordination states under the premise of keeping the total Cr^3+^ content consistent (Figure S26a, Supporting Information). The S‐ML intensity can be controlled by doping hetero‐valent cations of the same concentration (Figure S26b, Supporting Information). Small‐radius cations are more effective in replacing Mg^2+^ sites in the MgO host, reducing Cr^3+^ cluster and increasing Cr^3+^ ion pair (Figure S26c,d, Supporting Information). Clearly, there is a one‐to‐one correspondence between the formation energy and the intensity of EPR and ML, indicating that the S‐ML intensity is strictly dependent on the content of Cr^3+^ ion pairs. The charge balance within the MgO host also affects the ML and PL peak formats, with larger valence differences resulted broader peaks^[^ ^47^ ^]^ (Figure S26e,f, Supporting Information). Similar trends are observed in phosphors doped with different Li^+^ concentrations, further confirming the importance of Cr^3+^ ion pairs in S‐ML emission (Figure S27, Supporting Information). In contrast to non‐centrosymmetric materials like ZnS, LiNbO_3_ and CaZnOS, where internal piezoelectricity drives electron transfer transition to produce S‐ML,^[^ ^48^ ^]^ MgO: Cr^3+^ exhibits local piezoelectricity and ferroelectricity due to crystal distortion and defects. Piezoelectric force microscopy (PFM) and ferroelectric tests reveal that MgO: Cr^3+^ exhibits weak ferroelectricity and enhanced piezoelectric response, which may be attributed to broken inversion symmetry^[^ ^49^ ^]^ (**Figure** **4**a; Figure S28, Supporting Information). Owing to the existence of a local piezoelectric field, MgO: Cr^3+^ shows stronger steady‐state voltage and current outputs under longitudinal force, which is consistent with the working principle of PENG^[^ ^50^, ^51^, ^52^ ^]^ (Figure 4b; Figure S29, Supporting Information). Therefore, we focus on solving the electron transfer mechanism in the S‐ML optical part. The local piezoelectricity and ferroelectricity are derived from the crystal field distortion, so we have further exhibited the change of bond‐length and local electron clouds after Cr^3+^ doping. Owing to the hetero‐valent Cr^3+^ ions substitution, the Mg‐O bonds in [MgO_6_] octahedron are shortened compared to MgO (Figure 4c; Equation S1, Supporting Information). The slight change of Mg─O increases the local [MgO_6_] octahedral distortion, which is conducive to inducing the cooperative Jahn‐Teller effect to produce the S‐ML emission.^[^ ^53^ ^]^

Furthermore, DFT calculations and experimental results reveal that Cr^3+^ doping induces local crystal field distortion and electron cloud rearrangement, which reduces Cr^3+^ local symmetry and promotes energy conversion efficiency from force to light (Figure 4d; Figure S30, Supporting Information). Additionally, the distortion of the local environment around the Cr^3+^ ion can also be proved by the crystal orbital hamilton population (COHP) analysis to further to quantify the bonding strength of atomic pairs^[^ ^54^ ^]^ (Figure 4e). A positive value of −COHP is the bonding state, and a negative value indicates the antibonding state. The larger value of −COHP intends stronger covalent bonding. Compared to MgO, the Mg─O bond shows the positive values of −COHP curves near the Fermi energy after the formation of V_Mg_ defect, which is conducive to the transfer transition of electrons from defect to Cr^3+^ ion states.^[^ ^55^ ^]^ In addition, our focus is on the electronic modulations induced by defects and external strains to further understand the S‐ML mechanism. Clearly, it is a direct bandgap material with a forbidden band of 4.735 eV at the G point of MgO, in which the wide bandgap structure provides accommodation space for defect energy levels and multiple coordination Cr^3+^ ion states (Figure 4f). Apparently, the contribution of valence band maximum (VBM) comes from the electronic states of O 2p orbitals, and the Mg 2p/3d orbitals dominate the conduction band minimum (CBM). The introduction of defects cannot be avoided during the synthesis of oxides, which not only supplies defect states but also affects the electronic structures of nearest neighboring sites. After the introduction of Cr^3+^ and Li^+^ ion, the projected partial density of states (PDOS) is downshifted with a slightly reduced band gap due to the formation of mid‐level states (Figure 4g; Figure S31, Supporting Information). Notably, the mid‐level states will be generated near the Fermi level (E_f_) to narrow the band gap, which is conducive to the transition of electrons and energy storage of electrons. In addition, the electrons are enriched around the V_O_/Cr_Mg_ vacancy at CBM when an oxygen atom is lost, whereas the cation V_Mg_ vacancy is located at VBM to capture the hole defect. Moreover, the defect states will affect the electronic structure of neighboring atoms. So, the PDOS of O‐2p states for different models are demonstrated in Figure S32a (Supporting Information). The O‐2p orbitals affected by the near V_Mg_ and V_O_ vacancy have induced the empty states slightly above the E_F_ as the mid‐level defect states, which makes it possible to transfer electrons in the mid‐level defect states, further causing a potential electron tunneling effect. Notably, these defect states are similar position to the Cr‐3d orbitals, which allows the fast electron tunneling from these defect states to Cr ion sites. Meanwhile, the abundant electron states promote the electron excitations under external mechanical stimuli. For the absorption spectra, the formation of defects (V_Mg_ and V_O_) enables a strong absorption peak at 240–700 nm with a large improvement in the absorption intensity (Figure S32b, Supporting Information). It is noted that the absorption peaks slightly blue‐shift when an external compressive force is introduced (10 GPa), which is conducive to improving the S‐ML performance.

Thermoluminescence (TL) curves from 77 to 600 K show no significant peaks, indicating that defects do not participate in persistent luminescence but contribute to S‐ML (Figure S33a, Supporting Information). According to the analysis results of XPS defect content, the anionic defect (V_O_) is also easy to form at high temperature, even in air atmosphere^[^ ^56^ ^]^ (Figure S33b, Supporting Information). Combined with the calculation of formation energies of different defects, the V_Mg_ vacancy shows the lowest energy costs after Cr^3+^ ion doping (Figure 4h; Figure S34, Supporting Information). The formation energy of V_Mg_ vacancy is significantly affected by its positions, which can be divided into two types: V_Mg_‐(001) and V_Mg_‐(110). The high defect formation energy does not participate in the ML process, while the low defect formation energy does. Hence, the recombination of electrons and holes in the anionic/cationic defects is the main cause of S‐ML under mechanical stimuli. According to the above DFT calculation and experimental results, a S‐ML‐response model with optical and electrical output was proposed by a potential electron transfer process (Figure 4i). The electrical signal is derived from the interface‐triboelectric and local piezoelectricity effect between inorganic phosphor and organic elastomers, which is conducive to promoting the enhancement of S‐ML signal.^[^ ^57^ ^]^ Notably, the S‐ML mechanism is similar to that in PL, the main difference is where the origin of electrons and the transfer ways. Unlike the contact‐separation‐induced ML model, the local piezoelectric effect induced by local distortion is the key to the generation of S‐ML. The local piezoelectric field causes the piezoelectric polarization charge carries under external mechanical stimuli, further influencing the separation of the electron‐hole pairs, where the tilt of the energy band accelerates this process.^[^ ^58^ ^]^ Subsequently, the recombination of electrons and holes produces energy to activate Cr^3+^ luminescent center via a non‐radiative transition process, where the V_Mg_ and V_O_/Cr_Mg_ defects located in VBM and CBM play a role in storing holes and electrons. Meanwhile, the introduction of Cr^3+^ ions and defects will generate the mid‐level trap states within the band gap as the intermediate states, which is conducive to the fast electron tunneling from these gap states to Cr sites to produce the excellent S‐ML emission. In general, the excellent self‐powered photo‐electric response capability on MgO: Cr^3+^ phosphor is a necessary condition to be a tactile sensor.

### 2.5 NIR Temperature Sensing and Self‐Powered Stress Visualization

Combining the flexible sensing, bioimaging, and NIR emission of MgO: Cr^3+^, we creatively designed a flexible in situ force/temperature sensor that can quickly sense the external complex environment. The temperature sensing signals are obtained by using an FIR flexible optical fiber core and double‐cladding technology (Figure S35, Supporting Information). The data of the optical fiber sensor can be obtained by a self‐developed smart temperature sensing platform (Figure S36, Supporting Information). The temperature‐dependent PL spectra from 273 to 373 K were obtained, and then the corresponding FIR value can be calculated by the transition of ^2^E_g_→^4^A_2g_ (isolated Cr^3+^) and ^4^T_2g_→^4^A_2g_ (Cr^3+^ pairs) to further decouple the temperature information (Figure S37a,b, Supporting Information). To better describe the temperature measurement accuracy, we repeat the test at a fixed temperature (37 °C, Optimum human body temperature) and convert the FIR value to the corresponding temperature. Clearly, the detection limit was approximately ±0.5 °C between the FIR fiber sensor and the standard temperature, which indicates that the sensor has relatively good precision and anti‐disturbance performance (Figure S37c–e, Supporting Information). Moreover, the temperature sensor displays good reproducibility and fatigue resistance under ten consecutive cycles of temperature testing from 0 to 80 °C (Figure S37f, Supporting Information). Compared to the traditional temperature measuring devices, the flexible optical fiber sensor not only shows the real‐time, in situ, and flexible characteristics, but also responds quickly and stably to various temperatures.

**Figure 5:**
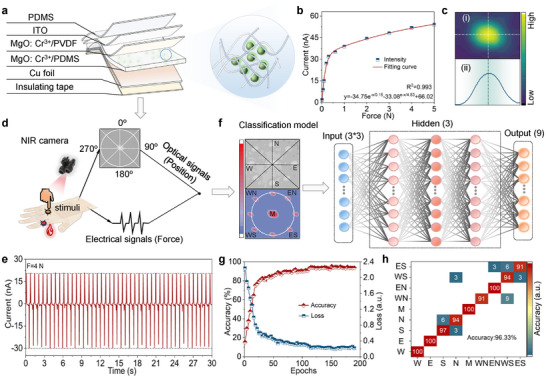
Potential application for NIR smart sensing and self‐powered stress visualization. a) Multi‐layer structure sensor: the MgO: Cr^3+^/PDMS and MgO: Cr^3+^/PVDF were encapsulated by Cu film and Indium‐Tin Oxide (ITO) film. b) The stable current output for MgO: Cr^3+^ and corresponding dotted line plot with force from 0.01 to 5 N. c) The sensor with optical signal output. d) Schematic illustration of the in situ photoelectric output in a multi‐layer flexible structure and the corresponding application of a proof‐of‐concept tactile sensing system. e) Cyclic test of the biomimetic sensor under continuous mechanical stimuli (F = 4 N). f) Schematic for the S‐ML photos of nine different positions, and corresponding 2D spatial optical mapping (left) and schematic of ANN architecture (right). g) The identification accuracy and loss function of the ANN variation with the training epoch number. h) Confusion matrix showing the predicted results of nine different positions.

The force signals are obtained by a design of a multi‐layer structure sensor that promotes the simultaneous output of photoelectric signals to respond to mechanical information (**Figure** **5**a; Figure S38, Supporting Information). As the mechanical force increases, the self‐powered electrical signal increases simultaneously, which indicates that the multi‐layer sensor is sensitive to mechanical force response (Figure 5b; Figure S39, Supporting Information). The in situ photoelectric signal is recorded by a NIR camera and electrometer, respectively (Figure 5c,d). Importantly, the sensor also displays good stability and force response, which is conducive to the practical application of the device for different complex environments (Figure 5e; Figure S40, Supporting Information). Notably, the NIR S‐ML emission can not only avoid the interference of visible light, guaranteeing the sensor with higher spatial resolution and lower light scattering effect, but also provides potential guiding value for the in situ detection application in the biological field. The excellent mechano‐optical response capability is helpful for 2D spatial optical mapping, which promotes the spatial position recognition of the sensor, different from traditional electrical array sensing systems. The spatial information of force distribution was investigated using a sensor under the same mechanical stimuli, where the electrical data exhibit excellent agreement in nine direction regions, which guarantees precise decoupling of the applied forces.

Meanwhile, the multi‐location spatial mapping information can also be obtained by extracting 2D optical distribution from the captured ML images, which is conducive to the accurate identification of the location of the applied force (Figure 5f). More importantly, combining neural network learning with a sensor for perception is an effective way to accurately acquire different mechanical information. Through numerical optimization methods, the neural network can actively find the hidden feature descriptors in the signal during the training process, so to establish an accurate decision to judge the location information of the applied stress. Using a database based on 900 2D‐NIR ML photos of different positions, one can train an ANN with 9 input neurons, where feature descriptors are displayed by connections between input, hidden, and output layers, and 9 output neurons correspond to 9 different orientations (east, west, south, north, central, southeast, southwest, northeast, northwest). With the increase of model training epochs, the recognition accuracy increases and the loss function decreases correspondingly, implying that the sensor has accurate orientation recognition resolution (Figure 5g). Even for relatively similar ML orientation photos, the model can discern their differences and make correct predictions by comparing subtle distinctions across multiple dimensions. The confusion matrix between the predicted and target positions displays an excellent accuracy of 96.33%, indicating that the proof‐of‐concept tactile sensing system has excellent recognition performance for different stimulation positions under external mechanical action (Figure 5h). Based on the centrosymmetric NIR S‐ML MgO: Cr^3+^, the tactile sensors with self‐powered tactile‐visual multimodal signal outputs and perception for external force and temperature may be used by the future generation of robots.

## 3 Conclusion

In all, the development of a flexible tactile sensor is demonstrated based on Cr^3+^‐activated NIR S‐ML material (MgO: Cr^3+^), which enables rapid, in situ, and real‐time response to mechanical and temperature stimuli, as that of human skin. Notably, the S‐ML phosphors exhibit stable and reproducible photoelectric performance over 10000 times under continuous mechanical stimuli. The excellent mechano‐sensing performance of MgO: Cr^3+^ comes from the local piezoelectricity and triboelectricity induced by the local distortion under hetero‐valent substitution of Cr^3+^ ions. There are multiple Cr^3+^ ion luminescent centers (isolated Cr^3+^, Cr^3+^ pair, and Cr^3+^ cluster), where Cr^3+^ pair dominates S‐ML emission and contributes to temperature sensing via FIR principles. The ML performance is attributed to the activations of Cr^3+^ neighboring defects, which create mid‐gap states that promote fast tunneling electron transfer to the nearby Cr^3+^ states. These defect states in VBM and CBM also cooperatively improve the absorption intensities to benefit the S‐ML performances. By integrating the flexible optical fiber sensing and multimodal signal output, we further developed a multi‐layered tactile device that mimics the sensing mechanism of human skin. The combination of electrical signals for force quantification, optical signals for 2D spatial mapping, and FIR‐based temperature detection enables comprehensive tactile sensing. ANN algorithms were employed to achieve an interaction recognition rate of 96.33%, demonstrating the sensor's potential for smart robotics and human‐machine interfaces. This work advances the understanding of NIR S‐ML mechanisms in centrosymmetric crystals, establishes a structure‐function‐device relationship, and provides a blueprint for next‐generation self‐powered multimodal tactile sensing systems. The integration of material design, mechanism insights, and device engineering offers critical guidance for the development of intelligent sensors in robotics, wearable technology, and biomedical applications.

## 4 Experimental Section

Materials and synthesis, characterization, and DFT calculation details are detailed in the Supporting Information.

## Conflict of Interest

The authors declare no conflict of interest.

## Supporting information



Supporting Information

## Data Availability

The data that support the findings of this study are available from the corresponding author upon reasonable request.;
