# Supplementary material for: Synergistic Optimization Between Chromium Local Coordination States Toward Self‐Powered High‐Repeatability Near‐Infrared Mechanoluminescence
Source: Adv Sci (Weinh). 2025 Nov 7;13(4):e18364. doi: 10.1002/advs.202518364 (PMC12822457; doi:10.1002/advs.202518364)
Supplement: Supplementary file 1 — Supporting Information [file ADVS-13-e18364-s001.pdf]

Supplementary Information

Synergistic Optimization Between Chromium Local Coordination States towards Self-powered High-repeatability Near-infrared Mechanoluminescence

Yao Xiao[1,3], Puxian Xiong[2,3,], Gaochao Liu[1,3], Yongsheng Sun[1], Xuesong Wang[1], Pan Zheng[1], Enhai Song[1] and Jiulin Gan[1,*]*

1. State Key Laboratory of Luminescent Materials and Devices; Institute of Optical Communication Materials; Guangdong Engineering Technology Research and Development Center of Special Optical Fiber Materials and Devices; Guangdong Provincial Key Laboratory of Fiber Laser Materials and Applied Techniques; South China University of Technology, Guangzhou 510640, China.

2. Department of Electrical and Electronic Engineering, The University of Hong Kong, Hong Kong, 999077, China.

3. These authors contributed equally to this work.

*Corresponding author e-mail: P.X. Xiong: pxxiong@hku.hk, J.L Gan: msgan@scut.edu.cn

Experimental Section

Materials and synthesis Reagents: MgO (99.9%, Aladdin), Cr_2O_3 (99.99%, Aladdin), Li_2CO_3 (99.99%, Aladdin), Na_2CO_3 (99.99%, Aladdin), K_2CO_3 (99.99%, Aladdin), ZnO (99.99%, Aladdin), $CaCO_3$ (99.99%, Aladdin) and $SrCO_3$ (99.99%, Aladdin) were purchased and used as received. MgO and Cr_2O_3 were mixed and ground homogeneously in an agate mortar for 20 min, and powders were sintered at 1500 ºC for 6 h under air atmosphere. Afterwards, the sintered samples were cooled down to room temperature within the furnace and ground again for 5 min for subsequent use and characterizations. Three test methods are used to study the ML properties: (1) the as-sintered crystals (1 g) were mixed with epoxy resin (4 g) to manufacture a hard cylinder (thickness: 15 mm; diameter: 25 mm), and the compression is added to the pellet; (2) Composited ML film (10 × 10 cm) prepared by quickly molding the phosphor (2 g) with PET is used for friction ML test; (3) The as-sintered crystals were mixed into polydimethylsiloxane (PDMS) and sealed between two polyethylene glycol terephthalate (PET) sheets using a home-made photographic-plasticization process.

Preparation of the MgO: Cr^{3+}/PVDF film: A solution was prepared by dissolving 1 g MgO: Cr^{3+} phosphor and 1 g PVDF-HFP granules in 6 mL DMF, and then the obtained solution was well sonicated and stirred vigorously for 6 h. Subsequently, the solution was poured into a petri dish or predesigned mold and kept in an oven at 80 °C for 1 h. In this way, the MgO: Cr^{3+}/PVDF film could be easily peeled off from the petri dish or predesigned mold to get a freestanding film with a thickness of 1 mm.

Optical fiber fabrication: Optical encapsulant (OE 6550 two-part silicone elastomer made of methylphenyl siloxane) and polydimethylsiloxane (PDMS Sylgard 184 two-part silicone elastomer) were obtained from Dow Corning Corporation (Shanghai, China). The phosphor material is filtered with 500 mesh screens to achieve powder particle uniformity. In the fabrication procedure, OE with 1:1 mass ratio of base and curing agent was configured as the core precursor. The precursor of the inner cladding is OE solution mixed with MgO: Cr^{3+} phosphor at 10:1 mass ratio, and the outer cladding was PDMS. All the precursor solution was mixed evenly by mechanical stirring

for 0.5 h and degassed in vacuum. The Teflon tube is used as the mold to form the fiber core (100 °C, 2 h). After de-moulding, the inner cladding (100 °C, 2 h) and the outer cladding (90 °C, 40 min) were spun and cured by heat. The emission spectra were obtained by a fiber optic spectrometer (QE650 Pro, Ocean Optics).

Structural characterization: Crystalline phase was characterized using an X-ray diffractometer (XRD) (Rigaku D/max-IIIA) with Cu-Kα1 radiation (1.5405 Å; cathode voltage, 40 kV; current, 40 mA) in the 2θ range of 10 - 90° at room temperature. Rietveld refinement method (FullProf software package) was adopted to confirm the formation of the target phase. Crystal structure was redrawn on basis of MgO PDF#45-0946 using Fullprof software. The X-ray near-edge absorption (XANES) structure spectra (Cr K-edge) were collected at the Beijing Synchrotron Radiation Facility, the data collection was carried out in transmission mode using an ionization chamber for Cr foil, and in fluorescence excitation mode using a Lytle detector for sample-flu. All spectra were collected in ambient conditions.The EXAFS spectra were obtained by subtracting the post-edge background from the overall absorption and then normalizing with respect to the edge-jump step. Subsequently, the χ (k) data were fitted the fourier transformed to real (R) space using a hanning window (dk = 1.0 $Å^{-1}$) to separate the EXAFS contributions from different coordination shells. To obtain the quantitative structural parameters around central atoms, least-squares curve parameter fitting was performed using the ARTEMIS module of IFEFFIT software packages[1]. Scanning electron microscope (SEM) images were captured using a FEI Tecnai G2 F20 microscope. HRTEM observations, SAED patterns, elemental mapping distribution, and energy dispersive X-ray spectrometry (EDS) were obtained using an Oxford XPLORE microscope microscope. For the preparation of TEM samples, the phosphors were dispersed in ethanol, then dropped on a copper grid and dried on a hot plate, and then tested on the computer. X-ray photo-electron spectroscopy (XPS) was acquired using a model Axis Supra+ instrument (UK, Kratos Company). Electron paramagnetic resonance (EPR) measurements were carried out using an EMXPLUS X-band spectrometer (Bruker, Germany) at room and low temperature (100 K) before and after

450 nm laser irradiation. The element contents of Mg and Cr were measured by inductively coupled plasma atomic emission spectrometer (ICP-AES, America Agilent 720ES (OES)).

Optical and electrical characterizations: Room-temperature photoluminescence (PL) and photoluminescence excitation (PLE) spectra were recorded using an FLS1000 fluorescence spectrophotometer equipped with a continuous xenon lamp (450 W) as an excitation source and a liquid-nitrogen-cooled NIR photomultiplier tube as a detector. The temperature-dependent PL spectra were measured by the same device equipped with cryogenic liquid-nitrogen plant equipment (TC202 Orient KOJI). The fluorescence lifetime decay curves were also measured by the same FLS1000 instrument using a microsecond flash lamp (μF900) as the excitation source. Ultraviolet–visible–NIR transmittance spectra were collected using a Hitachi UH4150 spectrophotometer, in which $BaSO_4$ was used as the reference standard. The thermoluminescence (TL) glow curves were recorded using a low temperature thermoluminescence three-dimensional spectrometer (LTTL-3DS-1) from 100 to 600 K. ML spectra and force curves were recorded by a universal press machine (CMT1104) and a self-developed force application device, a photo-counting system consisting of a photomultiplier tube (PHOTOSENSOR MODULES H10722), a photomultiplier tube (PMT) and a computer in the dark condition. All NIR ML and PL images were obtained by an auxiliary night-vision monocular (ONV2+, Orpha, Germany). Piezoelectric force microscopy (PFM) was carried out on an atomic force microscope (Oxford -MFP-3D Origin+). The ferroelectric properties were tested by a Radiant Precision Multiferroic Materials Analyzer (Premier II) at room temperature. The stable voltage and current signal were tested by a digital electrometer system (6517B, Keithley, USA) and a computer under a self-developed force application device.

Calculation Setup: To study the structure well, we used the Vienna ab initio simulation package (VASP) codes to calculate the lattice structure and the electron cloud distribution based on the density functional theory[2]. A spin-polarized magnetic field was considered in each calculation. Mg was replaced by Cr in a 3×3×3 supercell of

MgO host to fit the experimental facts ($Mg_{108}O_{108}$), which indicates a doping concentration at 1%. The generalized gradient approximation (GGA) with the Perdew-Burke-Ernzerhof (PBE) functional was employed as the exchange-correlation potential. The cut-off energy Ecut of 400 eV was used throughout all the calculations. A gamma-centered 2×2×2 k-mesh grid in the Brillouin zone was applied to determine the self-consistent charge density using Monkhorst-Packscheme. The crystal lattice was fully relaxed until the atomic force was less than 0.03 eV/Å. The energy convergence criterion for self-consistent electronic calculation was set to 10^{-5} eV/atom. The formation energy defect in charge state q is defined the following equation[3]:

$$E^f[X^q] = E_{tot}[X^q]- E_{tot}[bulk]-\sum n_i\mu_i+qE_F+ E_{corr}$$

Where $E_{tot}[X^q]$ is the total energy originated from a supercell calculation containing the defect, and $E_{tot}[bulk]$ is the total energy for the perfect crystal using an equivalent supercell. The integer n_i represents the number of atoms in type i (impurity atoms or host atoms) that have been added to ($n_i > 0$) or removed from ($n_i < 0$) the supercell to form the defect, and the μ_i is the corresponding chemical potentials of these species (chemical potentials display the energy of the reservoirs with which atoms are being exchanged). The analog of the chemical potential for “charge” is given by the chemical potential of the electrons, i.e., the Fermi energy E_F. Finally, E_{corr} is a correction term that accounts for finite k-point sampling in the case of shallow impurities, or for elastic or electrostatic interactions between supercells. We present the charge state of a defect with a superscript q. For example: for a neutral defect, q = 0; if one electron is removed, q = +1; if one electron is added, q = −1, etc. Interestingly, only neutral defects can occur in a metal. However, the defect can typically assume various charge states in a semiconductor or insulator, complected through exchanging electrons with an electron reservoir, where the energy is the electron chemical potential or Fermi level E_F, conventionally referenced to the VBM in the host. The defect formation energy depends on the chemical potentials of the constituent atoms. In the thermodynamic equilibrium, the chemical potentials are constrained within the following relation:

$$\mu_{Mg}+\mu_{O}=\mu_{MgO}$$

where μ_{MgO} is the total energy of one formula unit of MgO. Because the materials were synthesized in air atmosphere (i.e., O-rich atmosphere), μ_O was first approximated by half the energy of O_2 plus the effect of temperature and force. The atomic chemical potentials of the other species may be further determined by thermodynamic equilibrium conditions of various secondary phases containing the species.

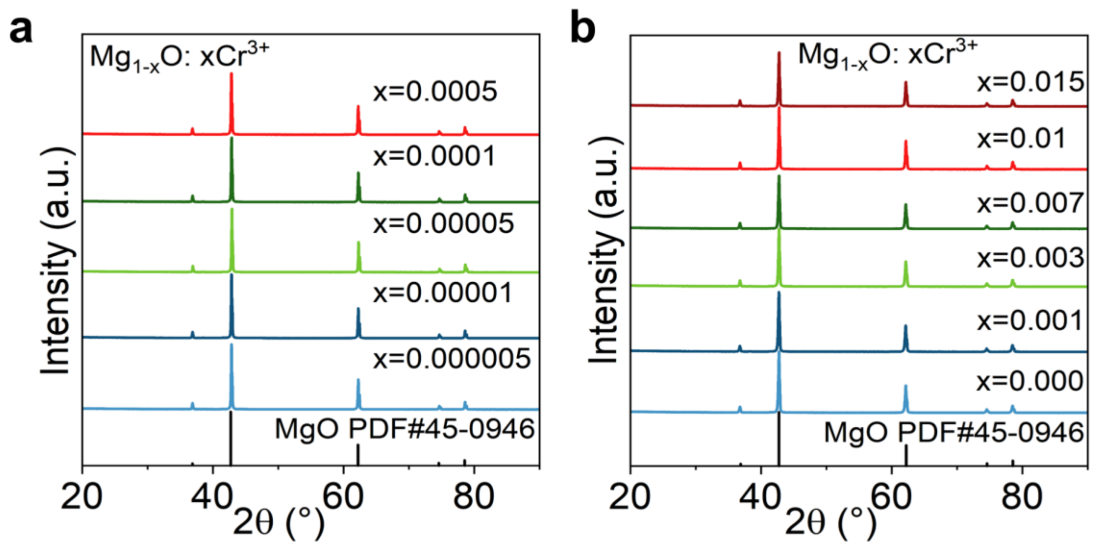

Figure S1. (a-b) XRD patterns of $Mg_{1-x}O$: xCr^{3+} (x=0.000005-0.015) and standard card of MgO (PDF # 45-0946).

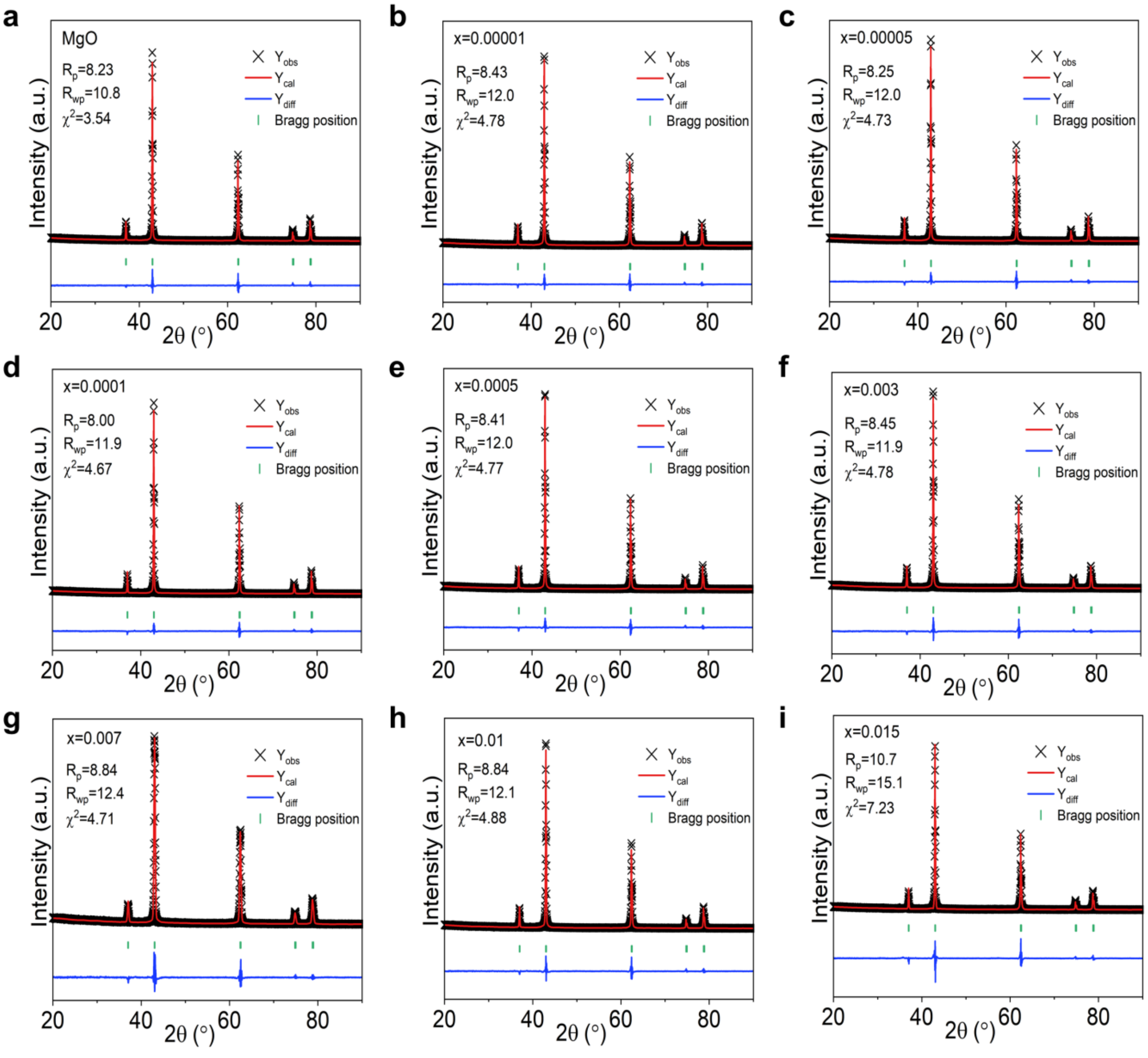

Figure S2. (a-i) Rietveld refinement results of $Mg_{1-x}O$: xCr^{3+} (x=0-0.015).

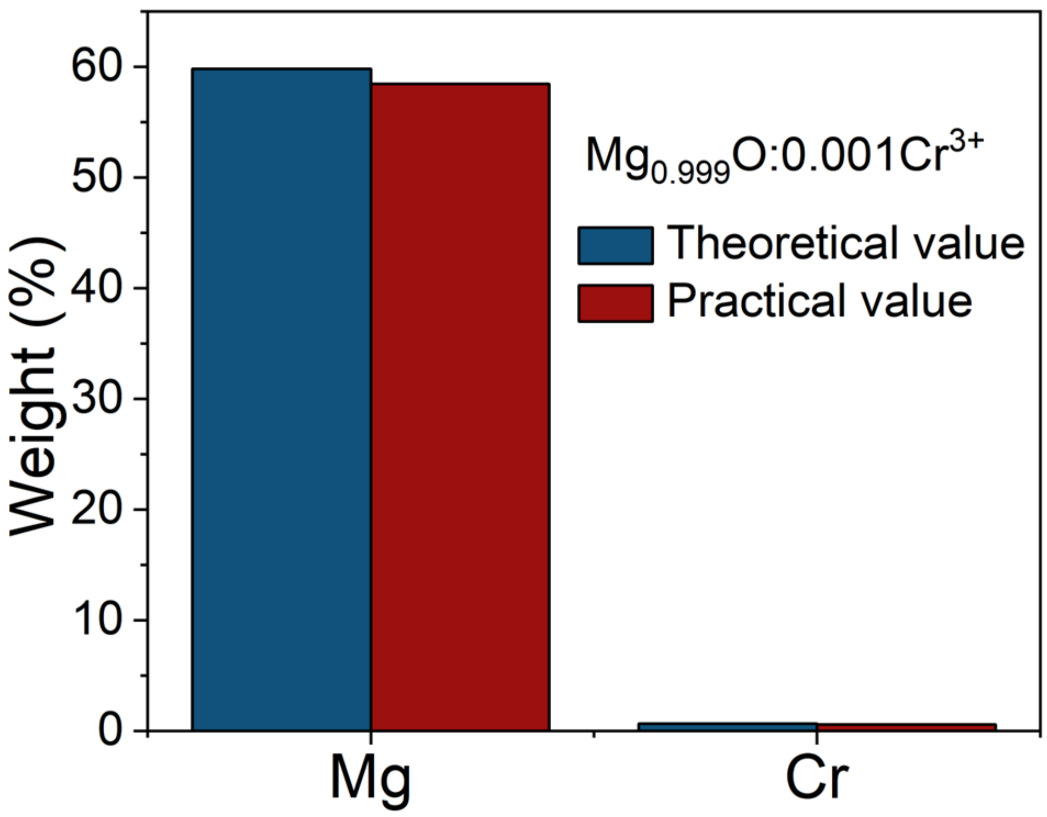

Figure S3. The ICP-AES elemental analysis of $Mg_{0.999}O$: $0.001Cr^{3+}$.

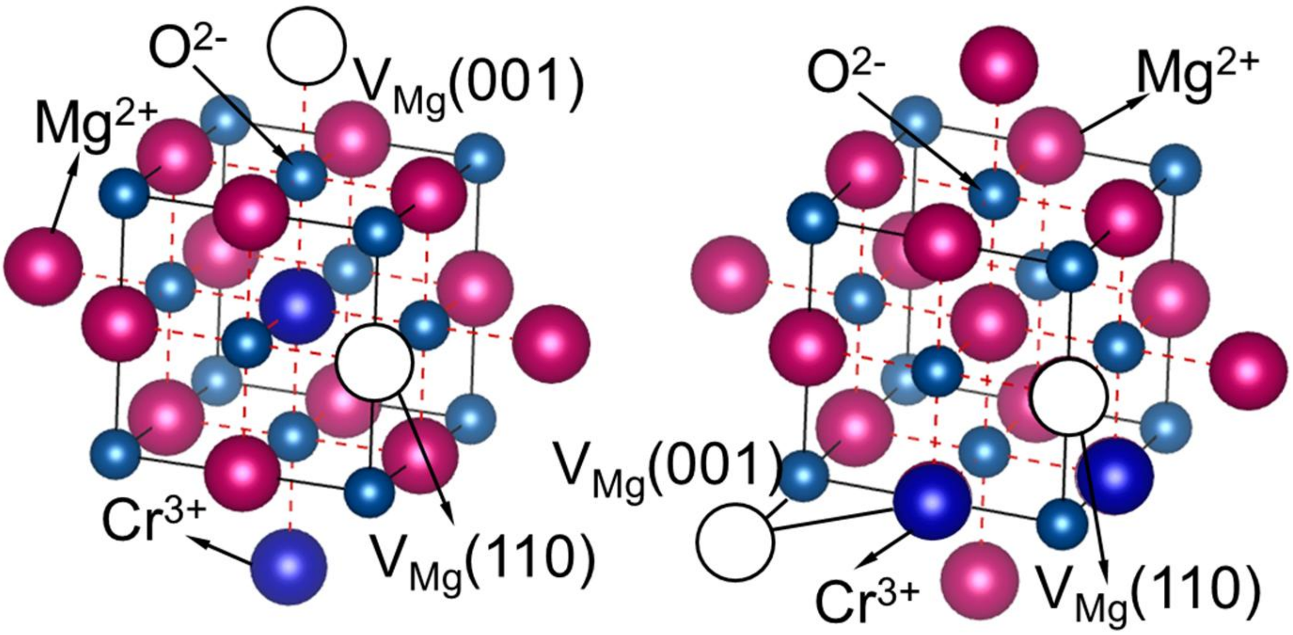

Figure S4. Cr^{3+} replaces different Mg^{2+} ion lattice sites to produce different types of V_{Mg} along the (001) or (110) crystal plane.

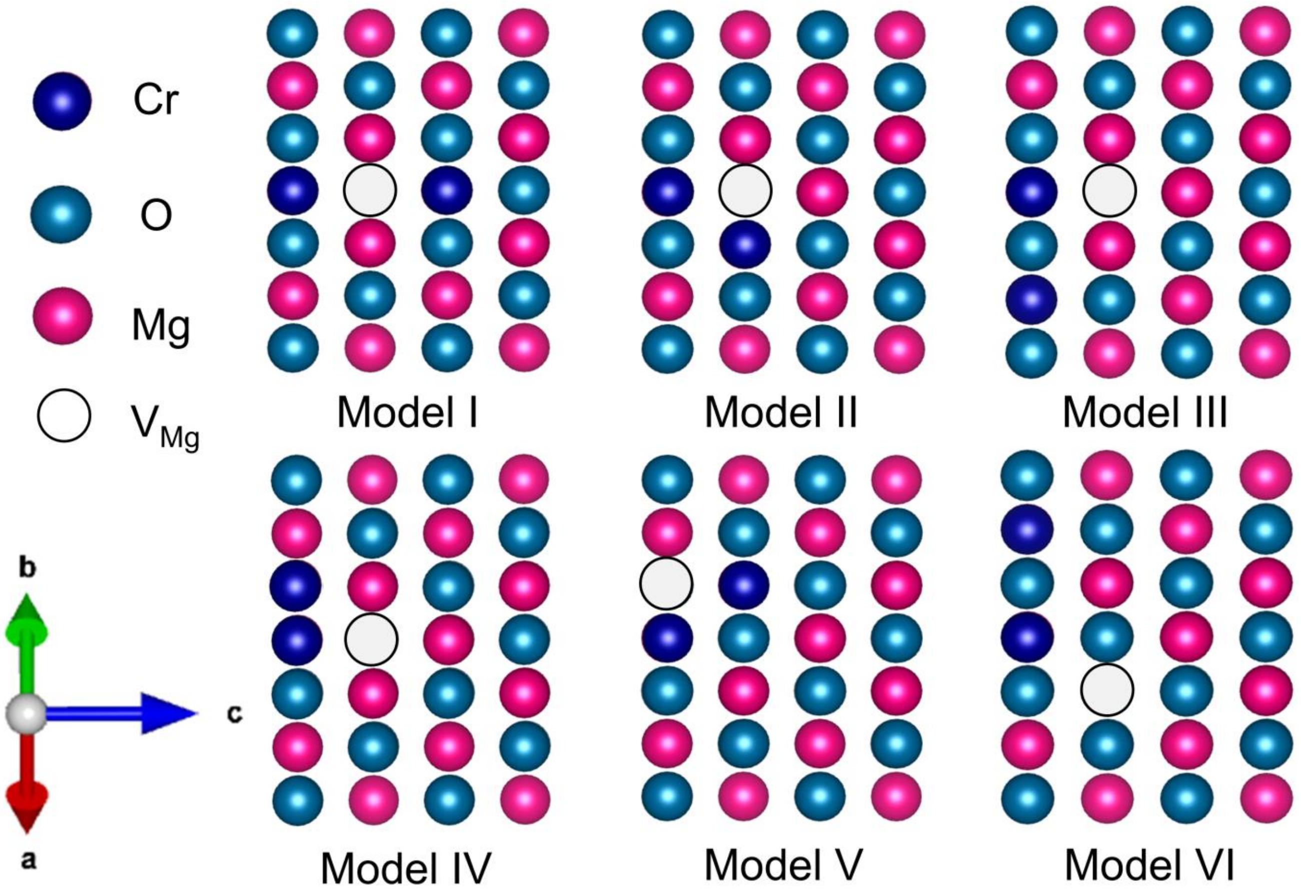

Figure S5. Six substitution models of MgO supercell with two Mg^{2+} ion replaced by two Cr^{3+} ion, while leaving a V_{Mg} vacancy after structure optimization.

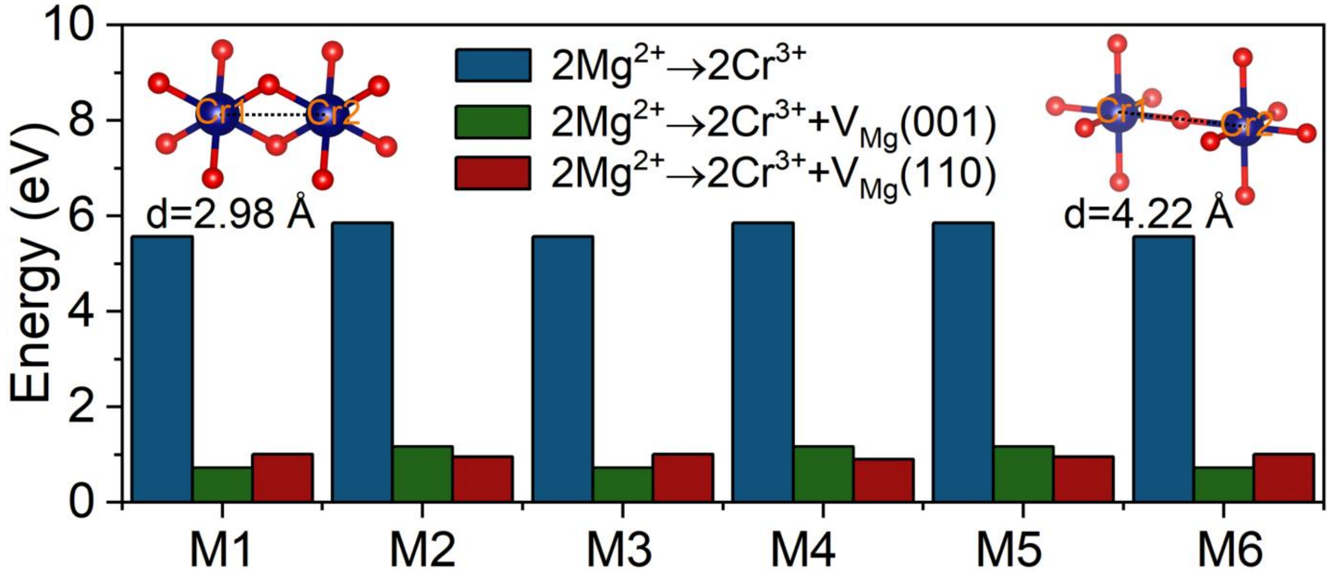

Figure S6. Defect formation energy for different substitution models. The distortion degree of $[MgO_6]$ can be evaluated by the distortion index (D) in **Equation S1**:

$$D = \frac{1}{n}\sum_{i=1}^{n}\frac{|I_i - I_{av}|}{I_{av}} \qquad (1)$$

Where I_i is the distance between the central atom and the *i* coordinated atom and I_{av} is the average bond length.

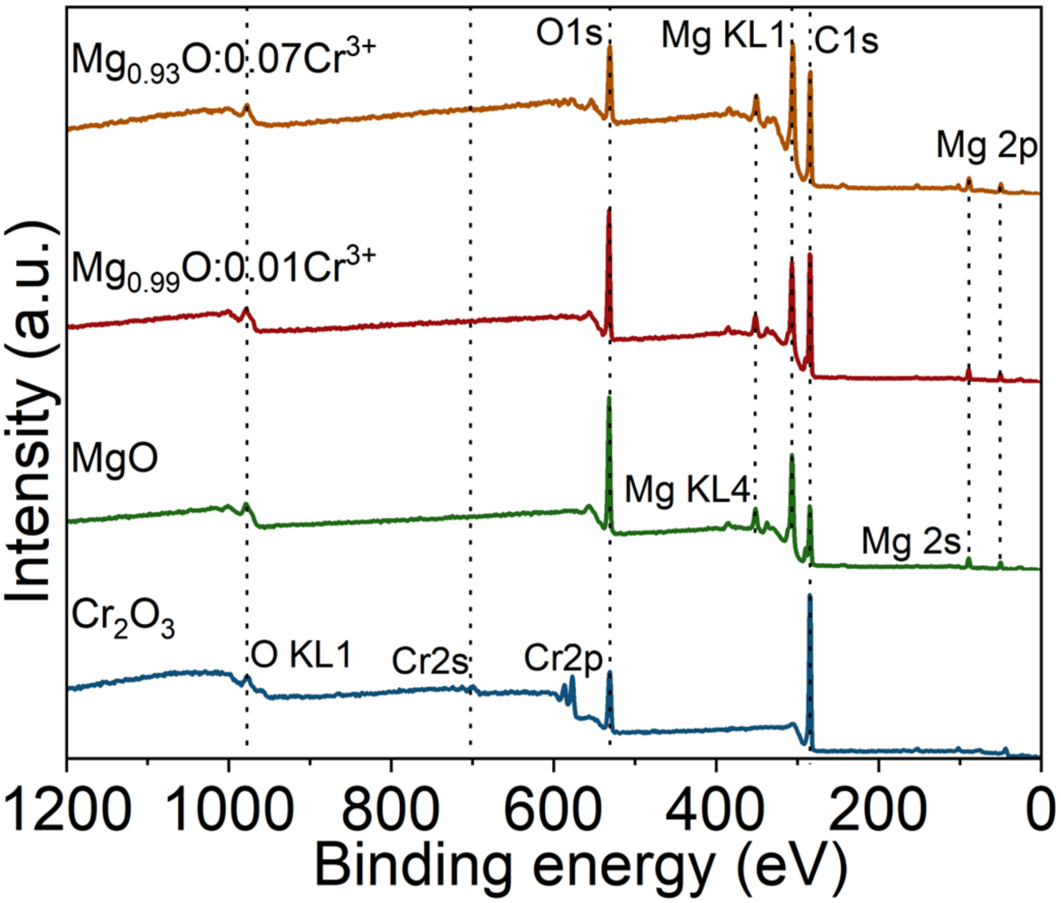

Figure S7. XPS survey scans of MgO: Cr^{3+} with Cr_2O_3 and MgO as the reference.

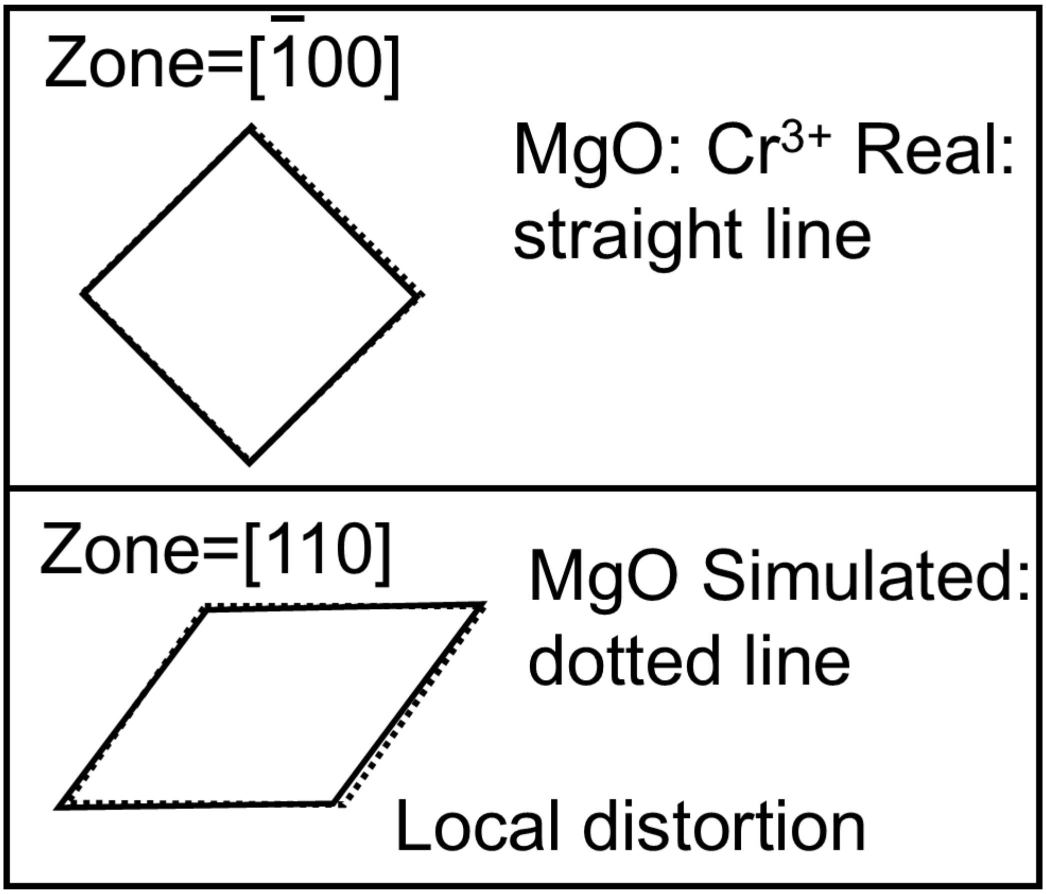

Figure S8. Comparison of the degree of distortion between real and simulated electron-diffraction patterns. The polygon formed by the lines between the different crystal plane is regarded as a whole, where the difference between the polygon before and after the real and simulation is defined as whether the local distortion in Cr^{3+}-doped MgO host occurs. There is no significant change in the overall MgO crystal structure due to a small amount of Cr^{3+} ion doping, there is a local crystal field distortion.

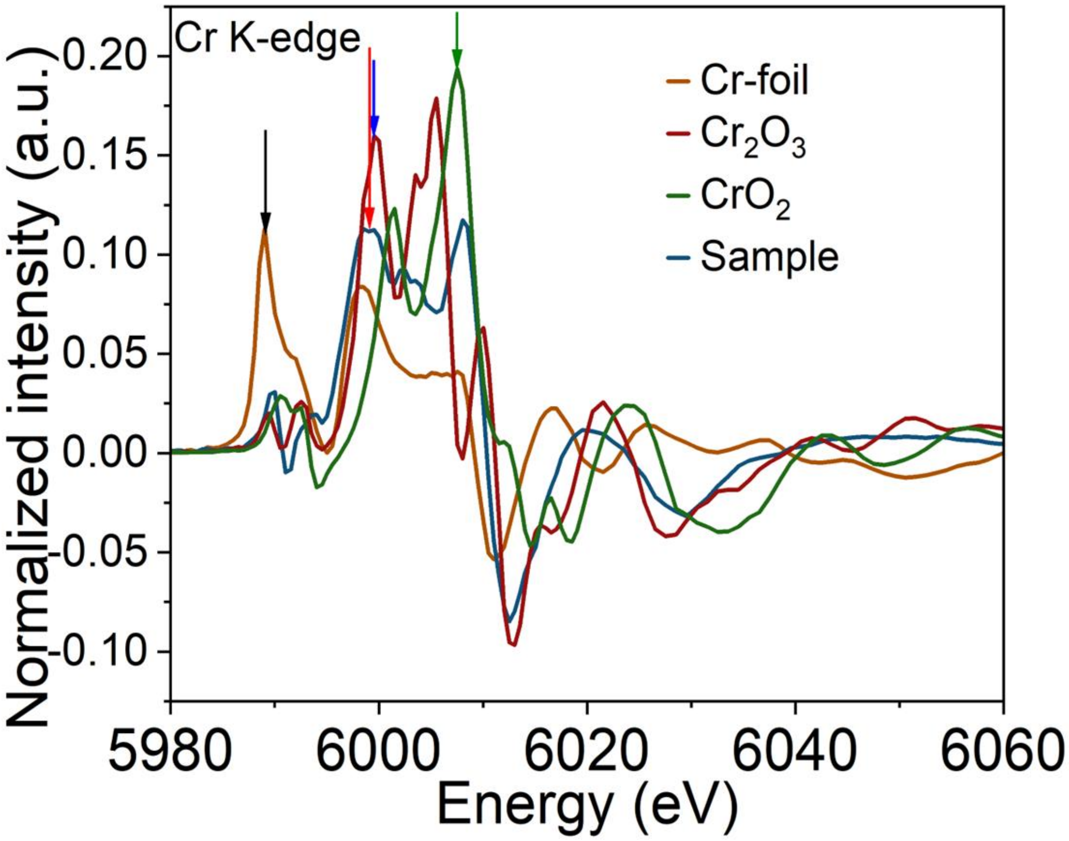

Figure S9. Cr K-edge XANES spectra of $Mg_{0.995}O$: $0.005Cr^{3+}$, Cr-foil, Cr_2O_3, and CrO_2.

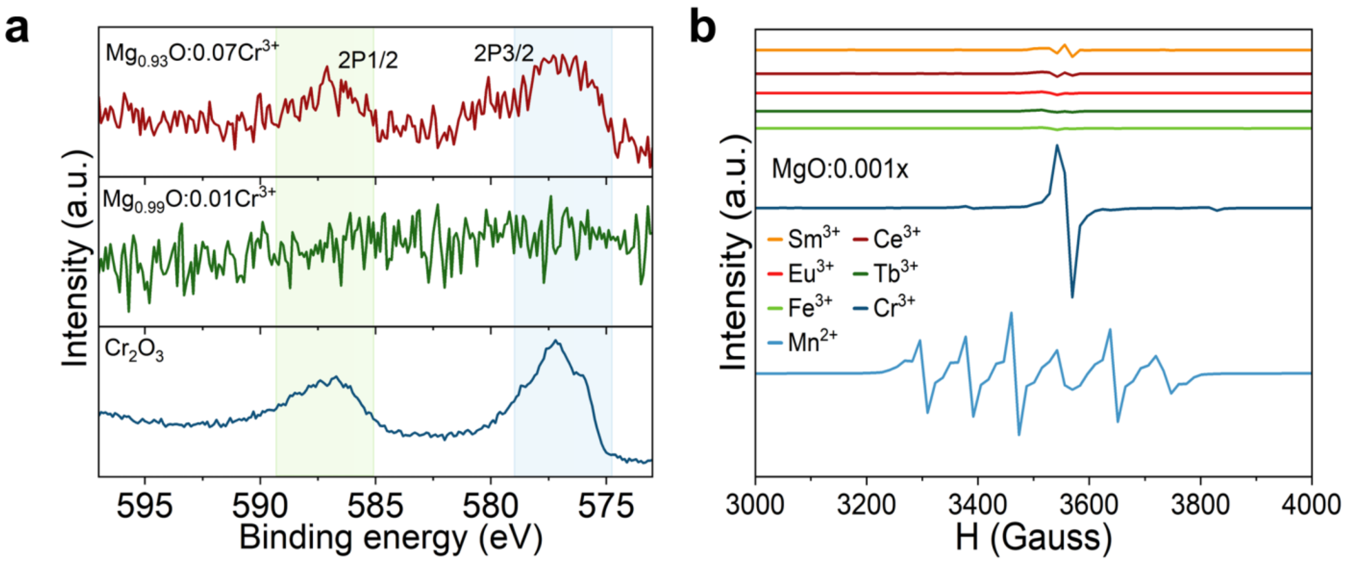

Figure S10. (a) High revolution XPS curves of Cr_2O_3, $Mg_{0.99}O$: 0.01Cr^{3+}, and $Mg_{0.93}O$: 0.07Cr^{3+}. (b) EPR curves of $Mg_{0.999}O$: 0.001x (x = Sm^{3+}, Ce^{3+}, Eu^{3+}, Tb^{3+}, Fe^{3+}, Cr^{3+} and Mn^{2+}).

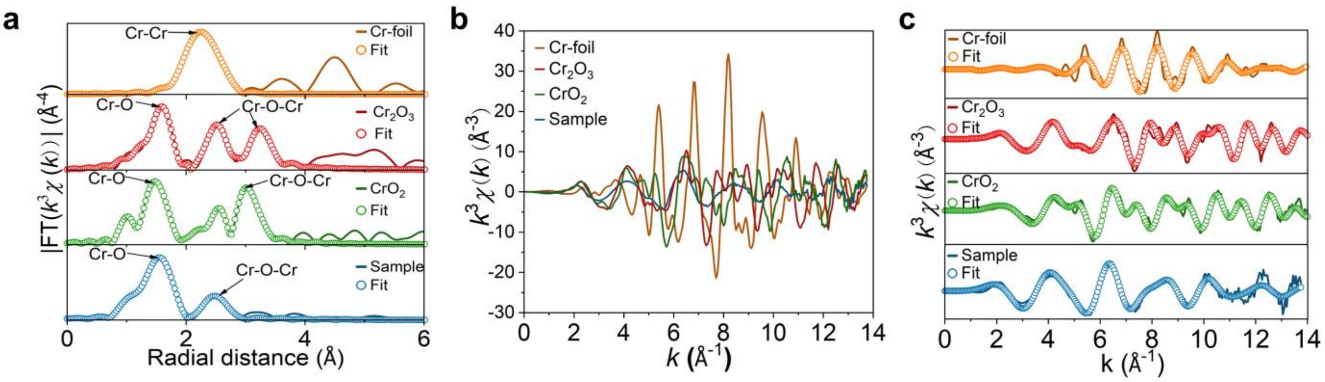

Figure S11 EXAFS and Fourier transform (FT) fitting curves in E-space of the $Mg_{0.995}O$: 0.005, Cr-foil, Cr_2O_3, and CrO_2. A multi-shell fit model was applied to determine the local structure of Cr^{3+} ion, and the R-factor is maintained in credible range. The detailed fitting parameters are summarized in Table S1. The coordination number (CN) for Cr^{3+} is 4.7 ± 0.4, which is close to 6 in MgO host, and the Cr-O distance is ~1.985 Å, indicating that Cr^{3+} occupied octahedral Mg^{2+} sites in this sample. Moreover, the fitting results also show that Cr^{3+} ions exist in the second coordination form, and the Cr^{3+}-O-Cr^{3+} distance is ~2.954 Å. It is closer to the shortest Mg^{2+}-Mg^{2+} distance (2.98 Å) calculated by DFT model, indicating that Cr^{3+}(Mg) would form the Cr^{3+} pairs by sharing with two oxygen ions. Meanwhile, the Cr^{3+} clusters may also exist under high-concentration Cr^{3+} ion doping. Additionally, the new emission band from the Cr^{3+} clusters in addition to the isolated Cr^{3+} and Cr^{3+} pairs emission can be expected in the samples doped with relatively high concentrations of Cr^{3+} ion.

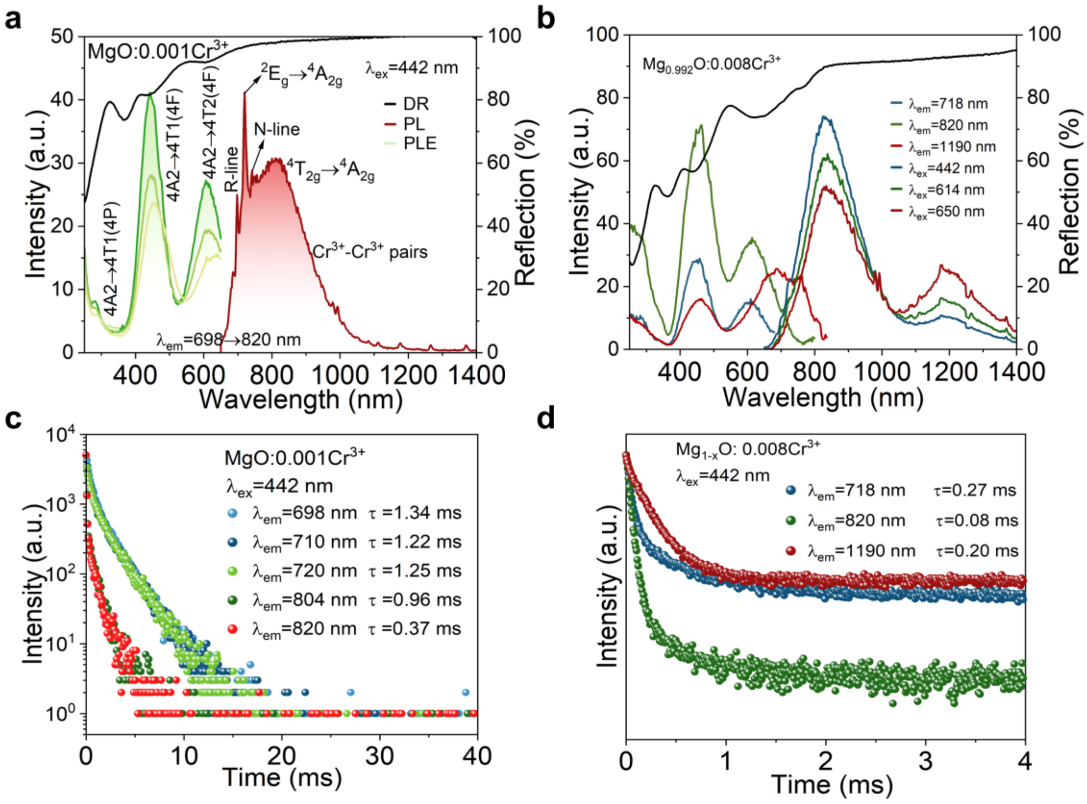

Figure S12. (a) PLE, PL and DR spectra of MgO: 0.001Cr^{3+}. (b) PLE, PL and DR spectra of MgO:0.008Cr^{3+}. (c) Fluorescence lifetime decay curves of MgO: 0.001Cr^{3+} under 442 nm excitation (PL peaks at 698, 710, 720, 804 and 820 nm). (d) Fluorescence lifetime decay curves of MgO: 0.008Cr^{3+} under 442 nm excitation (PL peaks at 718, 820 and 1190 nm). The different change trend between the fluorescence lifetime decay curves is due to the difference between the NIR and the visible monitoring probe, but this does not affect the actual value of fluorescence lifetime. All fluorescence lifetime decay curves can be fitted by the double exponential attenuation in **Equation S2 and S3**:

$$I(t) = I_0 + A_1 \exp(\frac{-t}{\tau_1}) + A_2 \exp(\frac{-t}{\tau_2}) \qquad (2)$$

$$\tau^* = \frac{A_1\tau_1^2 + A_2\tau_2^2}{A_1\tau_1 + A_2\tau_2} \qquad (3)$$

where $I(t)$ represents the intensity at time t, τ_1 and τ_2 are the short and long decay component, respectively, and A_1 and A_2 are constants. Clearly, the fluorescence lifetime maintained at the ms level is similar to typical Cr^{3+} ion emission[4]. Notably, the fluorescence lifetime monitoring at 1190 nm is 0.2 ms, which is much longer than that monitoring at 820 nm. Meanwhile, this decay lifetime is significantly longer than that of typical Cr^{4+} ion, which is only a few tens of us, which

further confirms that the NIR-II emission is unrelated to Cr^{4+} ion[5]. Moreover, all fluorescence lifetime decay curves are well fitted with the double-exponential function, indicating that there are multiple luminescent centers in Cr^{3+}-doped MgO.

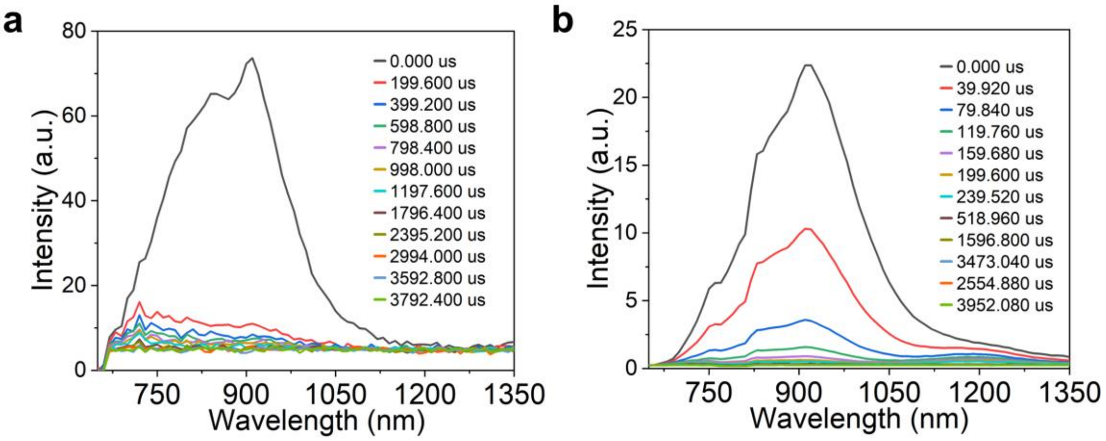

Figure S13. (a) Time-resolved PL spectra of $Mg_{0.999}O$: $0.001Cr^{3+}$ under 442 nm excitation. (b) Time-resolved PL spectra of $Mg_{0.992}O$: $0.008Cr^{3+}$ under 442 nm excitation. The Time-resolved PL spectra identify the multiple Cr^{3+} ion luminescent centers in MgO host. Meanwhile, the existence of a new luminescence center at 1190 nm was verified under high concentration of Cr^{3+} doping, and it does not participate in the energy transfer process.

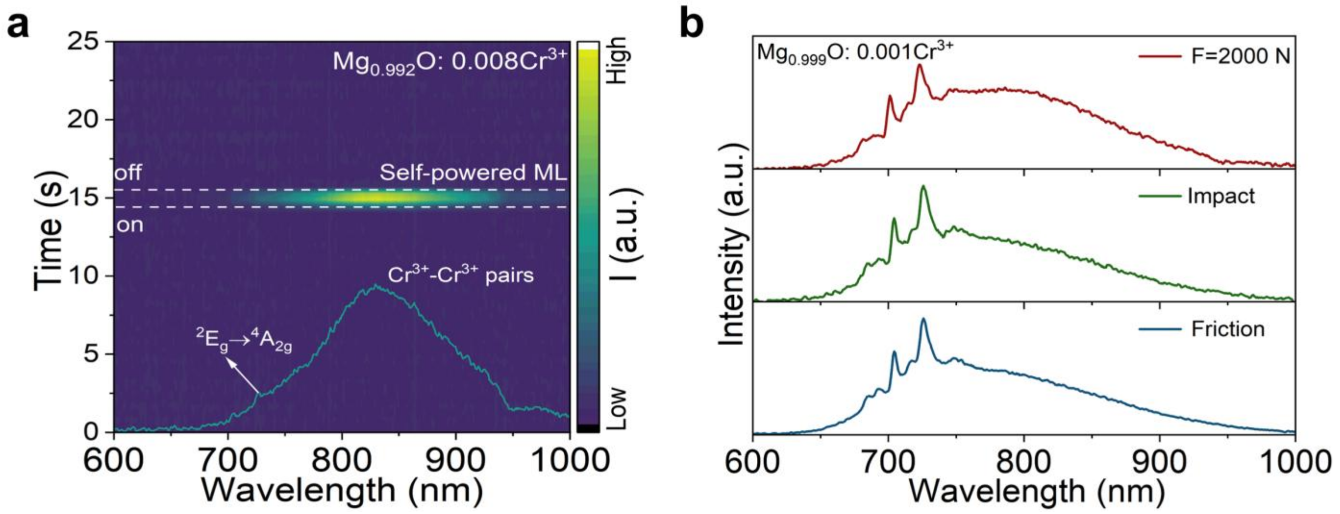

Figure S14. (a) 2D color map surface graph of self-powered ML of $Mg_{0.992}O$: $0.008Cr^{3+}$ recorded with time decay under 2000 N. (b) ML spectra of $Mg_{0.999}O$: $0.001Cr^{3+}$ under different mechanical stimuli conditions (Compression force (2000 N), friction (5 N), and impact force (5 N). To investigate the self-powered characteristics, the corresponding hard pellets were prepared by mixing MgO: Cr^{3+} powders with epoxy resin in appropriate proportions, and ML test was carried out under high loading (2000 N). On the contrary, the small pressure test is based on the PET/PDMS with phosphor composite film. The purpose of the selection of multiple ML test schemes is to investigate the luminescence mechanism inside the material and verify its potential application value in multi-modal tactile perception.

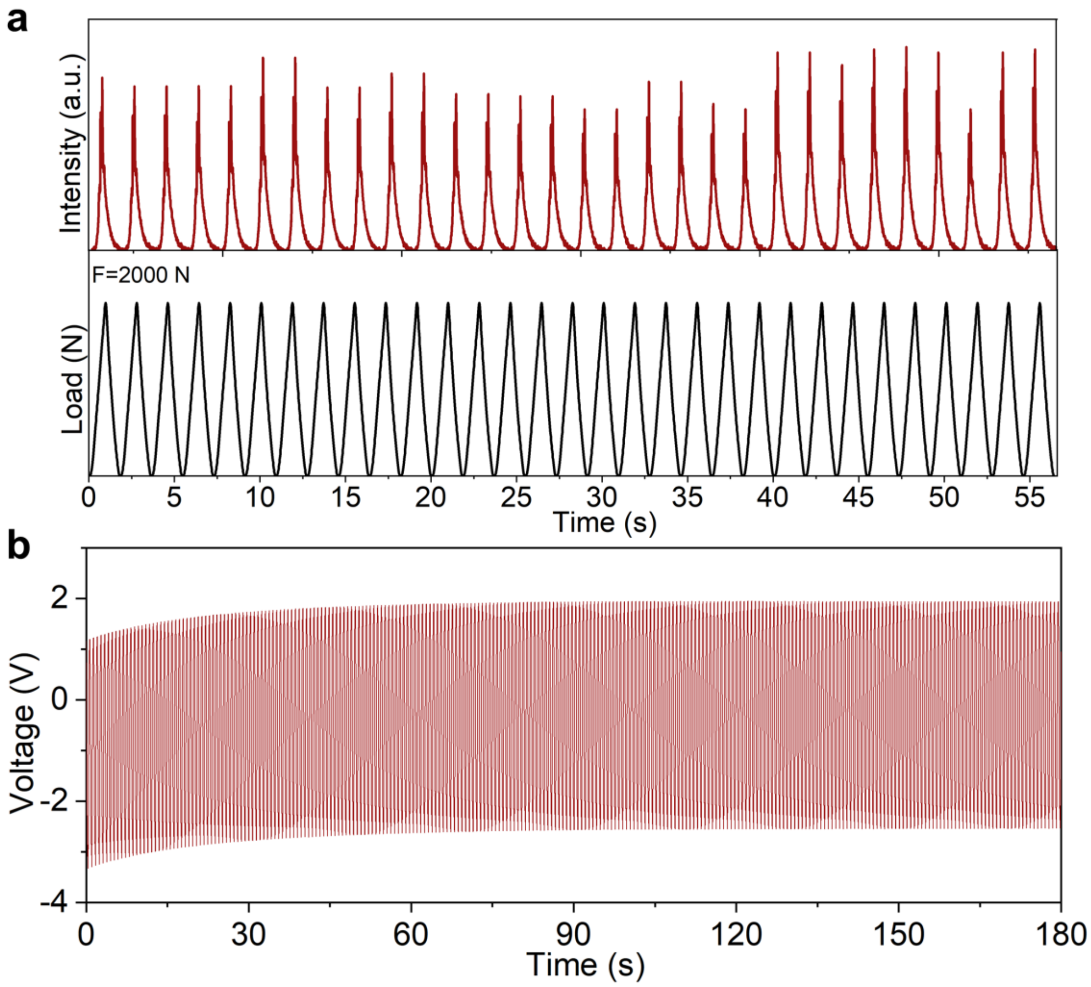

Figure S15. (a) S-ML cyclic test results of MgO: Cr^{3+} samples for 30 cycles under 2000 N. (b) Cyclic test of electricity under continuous mechanical stimuli (F = 3 N).

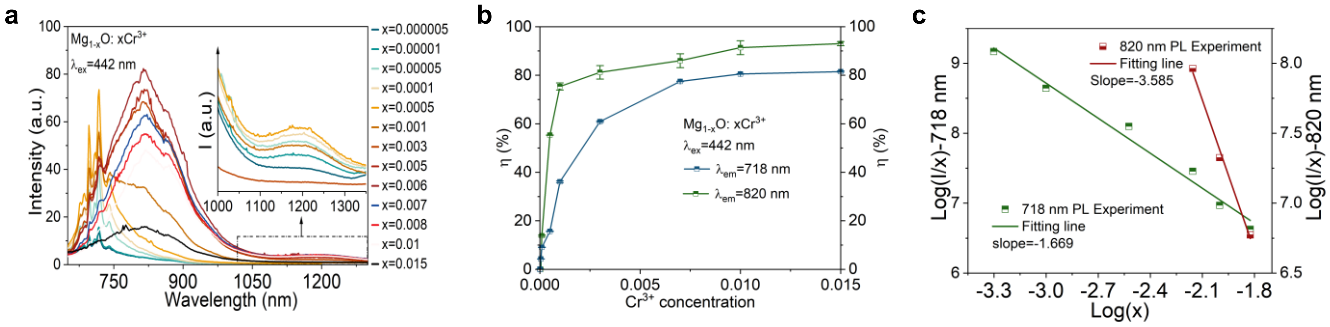

Figure S16. (a) PL spectra of $Mg_{1-x}O: xCr^{3+}$. (b) Energy transfer efficiency under different Cr^{3+} concentrations and the corresponding error bars. Energy transfer efficiency (η) of Cr^{3+} ion between different local coordination environment is evaluated by comparing the change rate of fluorescence lifetime in **Equation 4**[6]:

$$\eta = 1 - \frac{\tau}{\tau_0} \tag{4}$$

Where τ is the effective fluorescence lifetime for different Cr^{3+} ion concentration, and τ_0 is the effective fluorescence lifetime when the concentration of Cr^{3+} ion is x =0.000005. (d) Plot of *log(I/x)* versus *log(x)* for $Mg_{1-x}O: xCr^{3+}$. The concentration quenching is derived from non-radiative energy transfer of Cr^{3+} ion, which may occur via the super-exchange or dipole-dipole interaction. Hence, in order to confirm the concentration quenching mechanism, critical distance (R_c) is calculated in **Equation S5**[7]:

$$R_c = 2[\frac{3V}{4\pi X_c N}]^{\frac{1}{3}} \tag{5}$$

Where V is volume of the unit cell, X_c is critical concentration, and N is the number of available sites per unit cell that Cr^{3+} ion can occupy. According to the values of V = 74.5413 Å^3, X_c = 0.006, and N = 6, R_c can be calculated to be 15.8163 Å. Based on the Dexter theory, the critical distance is much greater than 5 Å, indicating that the effect of concentration quenching is mainly depended on an electric multipolar interaction[8]. The type of electric multipolar interaction can be clarified by the **Equation S6**[9]:

$$\frac{I}{x} = K[1 + \beta(x)^{\frac{\theta}{3}}]^{-1} \tag{6}$$

Where I is PL intensity, x is the concentration of doped ion, K and β are the constants for each type of interaction in given host lattice, and θ is the index of electric multipole corresponded to the dipole-dipole (θ = 6), dipole-quadrupole (θ = 8), and quadrupole-quadrupole (θ = 10),

respectively. Because the value of θ can be calculated by fitting the linear relationship between *Log(I/x)* and *Log(x)*, the slope *(-θ/3)* is around -1.669 and -3.585, respectively. Intuitively, corresponding θ is closer to 6 and 10, implying that concentration quenching mechanism and energy transfer pathway is due to the dipole-dipole (*d-d*) and quadrupole-quadrupole (*q-q*) interaction.

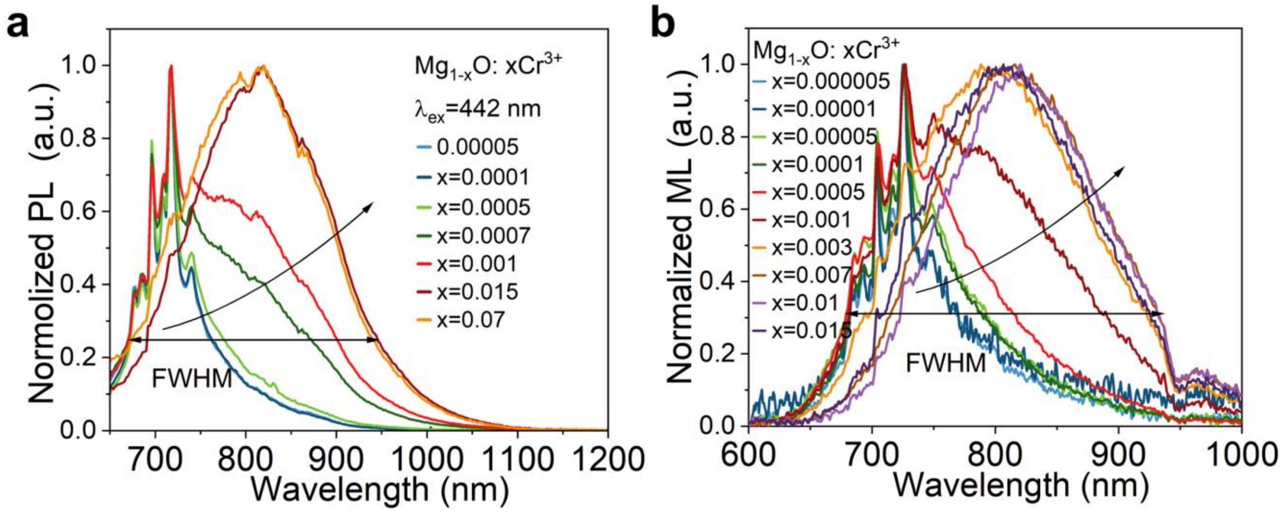

Figure S17. (a) Normalized PL spectra of $Mg_{1-x}O$: xCr^{3+} (x=0.00005-0.07) under 442 nm excitation. (b) Normalized S-ML spectra of $Mg_{1-x}O$: xCr^{3+} (x=0.000005-0.01).

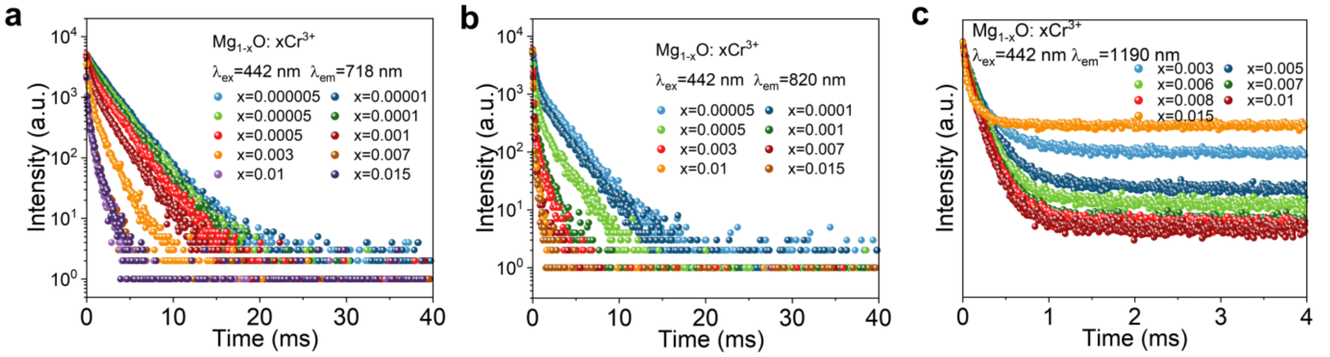

Figure S18. Fluorescence lifetime decay curves of $Mg_{1-x}O$: xCr^{3+} monitored at (a) 718 nm, (b) 820 nm and (c) 1190 nm under 442 nm excitation. The different change trend between the fluorescence lifetime decay curves is due to the difference between the NIR and the visible monitoring probe, but this does not affect the actual value of fluorescence lifetime.

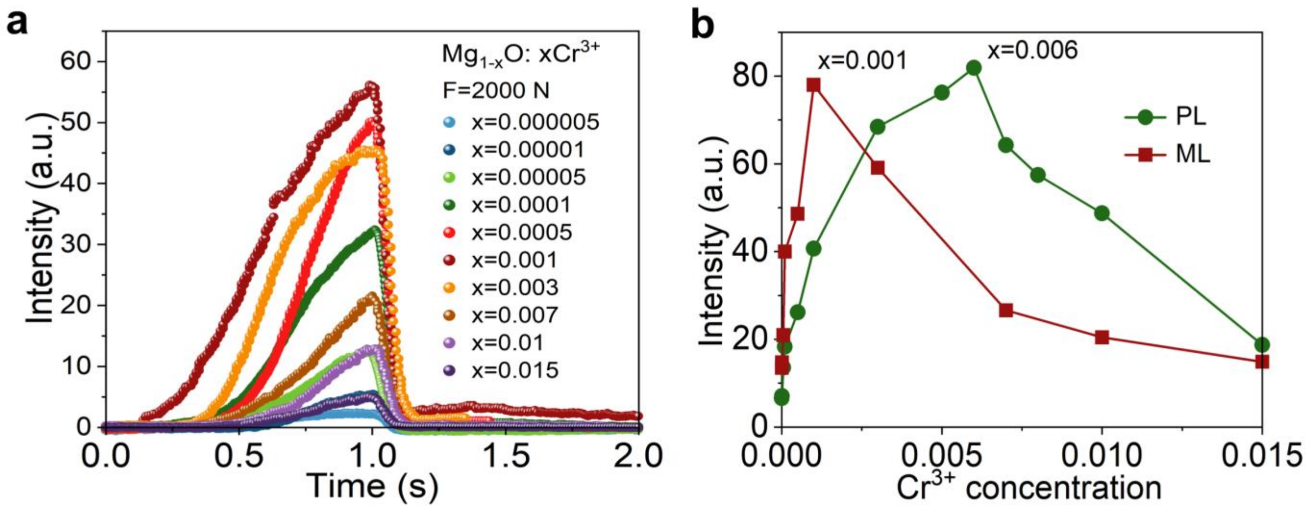

Figure S19. (a) S-ML intensity versus test time curves under 2000 N for $Mg_{1-x}O$: xCr^{3+} from 0.000005 to 0.015. (b) Integrated PL and ML intensity from 650 to 1400 nm for $Mg_{1-x}O$: $x Cr^{3+}$ from 0.000005 to 0.015.

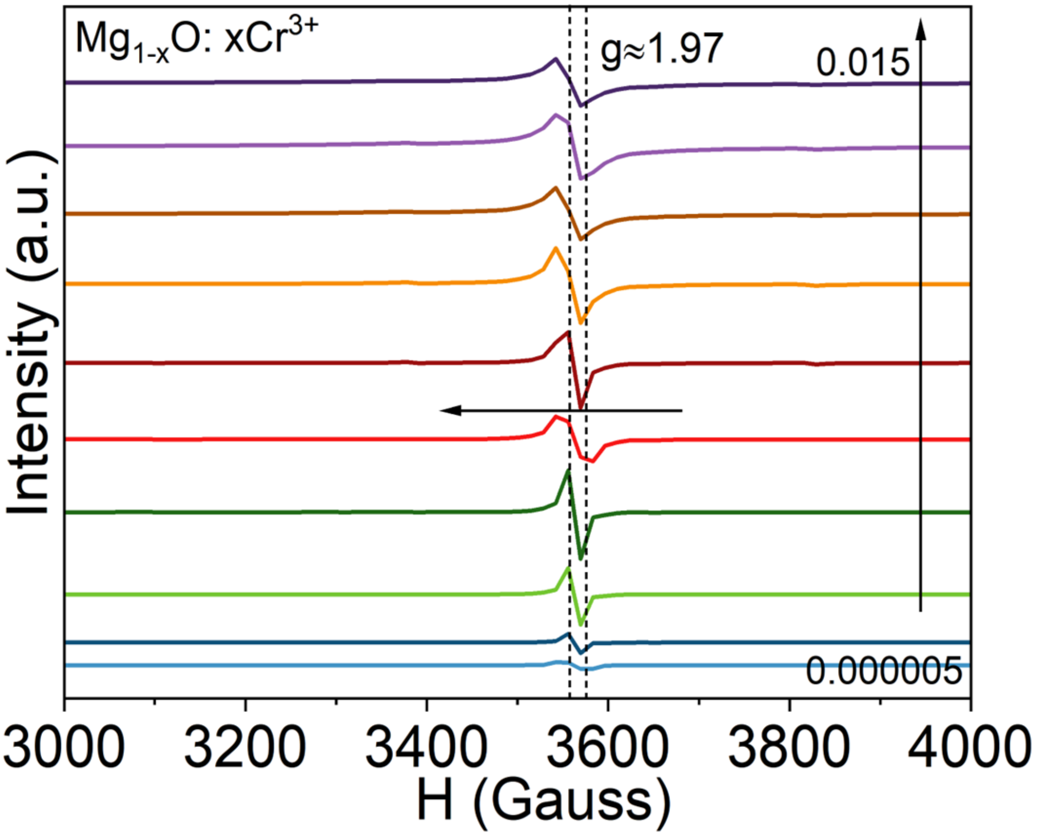

Figure S20. EPR curves of $Mg_{1-x}O$: xCr^{3+} from 0.000005 to 0.015.

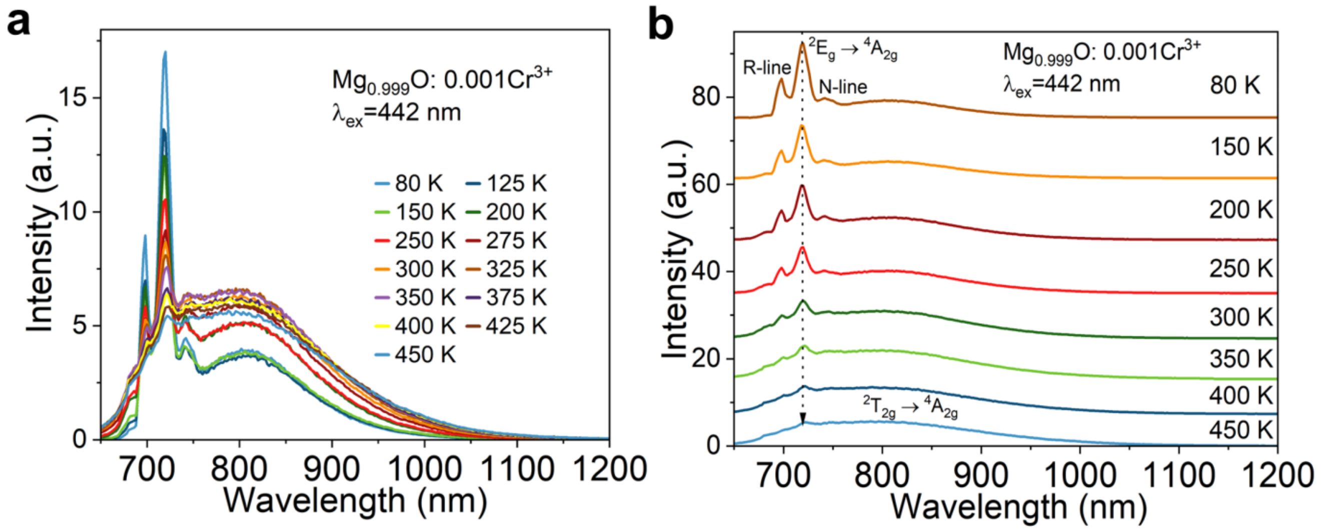

Figure S21. Temperature-dependent PL spectra of $Mg_{0.999}O$: $0.001Cr^{3+}$ from 80 to 450 K under 442 nm excitation.

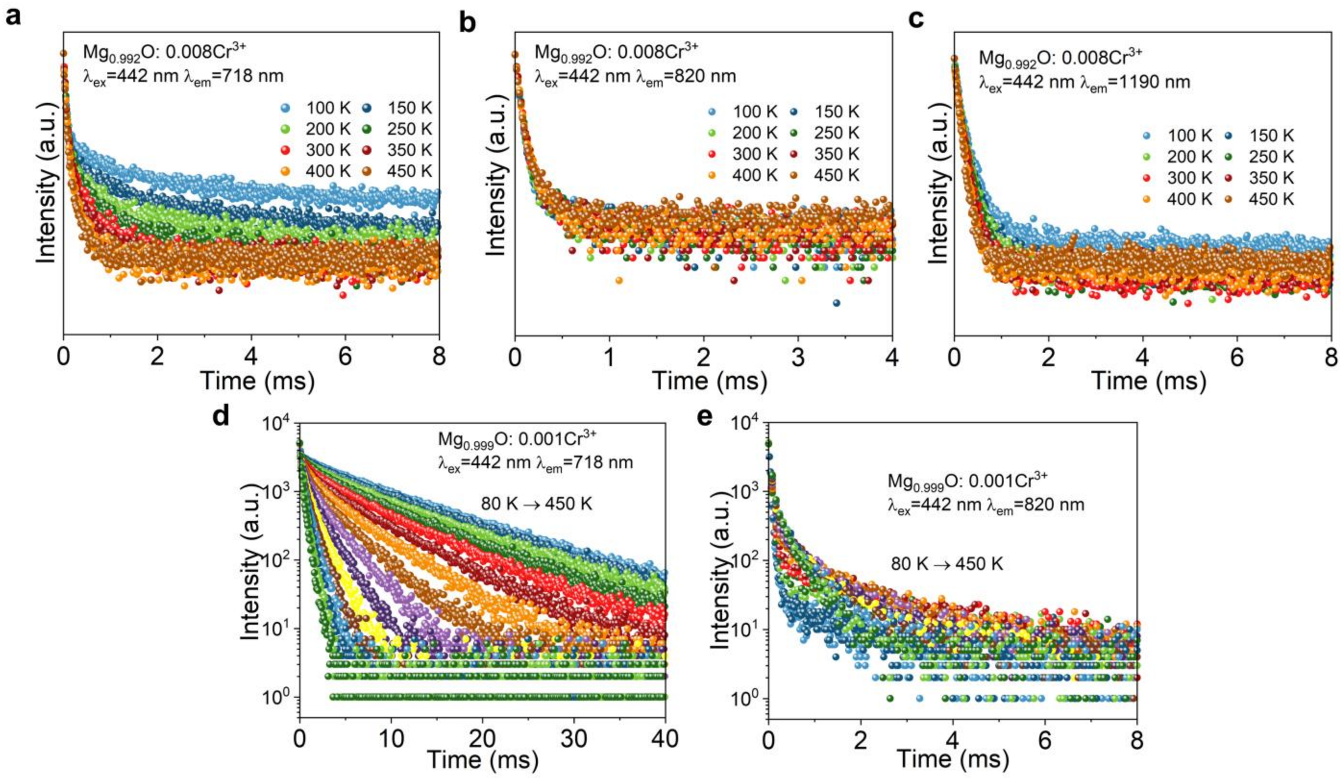

Figure S22. Temperature-dependent fluorescence lifetime decay curves of $Mg_{0.992}O$: $0.008Cr^{3+}$ from 100 to 450 K monitored at (a) 718 nm, (b) 820 nm and (c) 1190 nm under excitation by 442 nm light. (d-e) Temperature-dependent fluorescence lifetime decay curves of $Mg_{0.999}O$: $0.001Cr^{3+}$ from 80 to 450 K. The different change trend between the fluorescence lifetime decay curves is due to the difference between the NIR and the visible monitoring probe, but this does not affect the actual value of fluorescence lifetime.

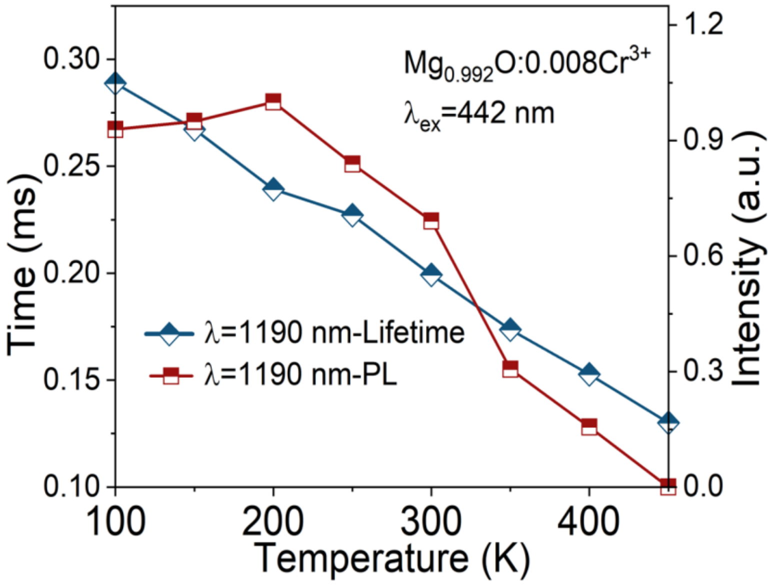

Figure S23. Integrated PL intensity and fluorescence lifetime from 100 to 450 K for $Mg_{0.992}O$: $0.008Cr^{3+}$. The Arrhenius formula is used to describe thermal quenching phenomenon in **Equation S7**[10]:

$$I(T) = \frac{I_0}{I + A\exp(-\frac{\Delta E}{KT})} \tag{7}$$

where I_0 is the initial integrated intensity, I_T is the integrated intensity at a given temperature, and k is Boltzmann constant. Relationship between *1/kT* and $Ln(I_0/I_T\text{-}1)$, as obtained by the linear fitting, where the slope represents the *ΔE* value. The *ΔE* represents the activation energy that electrons required to go from the lowest excited state to the intersection. Notably, the fitting results are divided into two parts, where one is the low temperature region with smaller slope and the other is the high temperature region with larger slope. According to previous investigations, the lower *ΔE1* represents the activation energy required for the non-radiative transition corresponding the C→D process in Figure 4f. The higher *ΔE2* is the activation energy required for thermal ionization corresponding the D→F process[11].

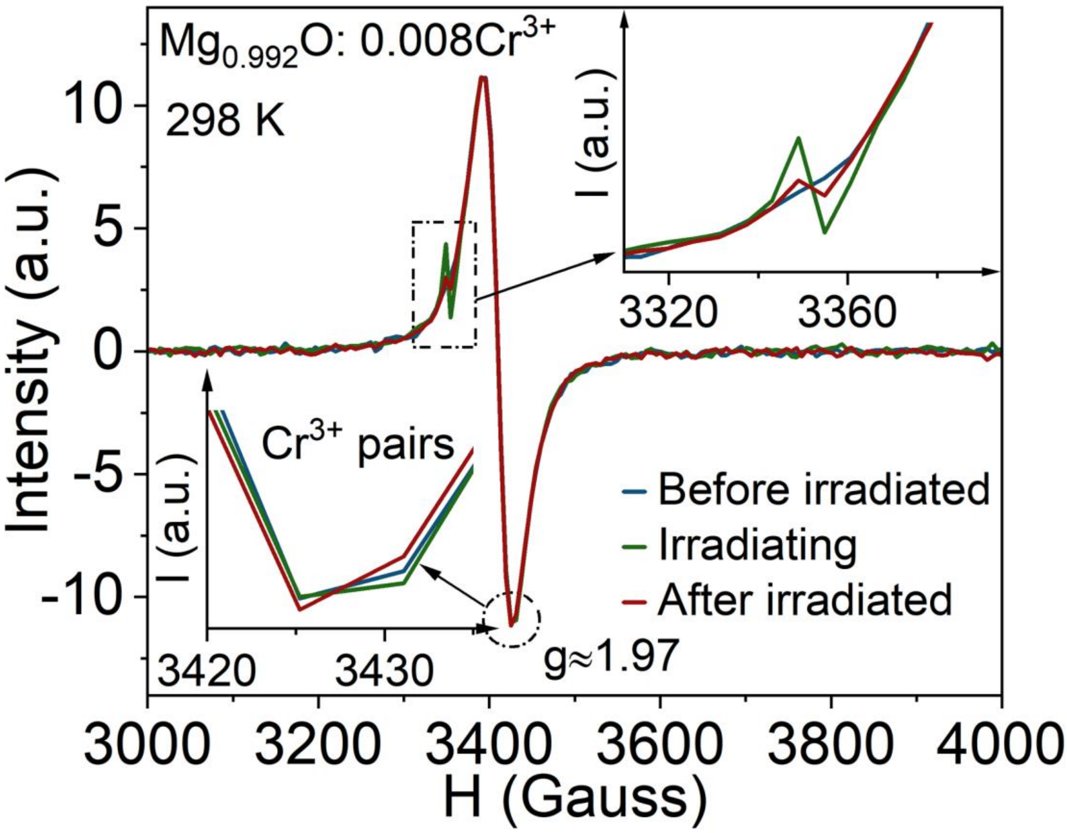

Figure S24. In-situ EPR curves of $Mg_{0.992}O$: $0.008Cr^{3+}$ with and without irradiation at room temperature.

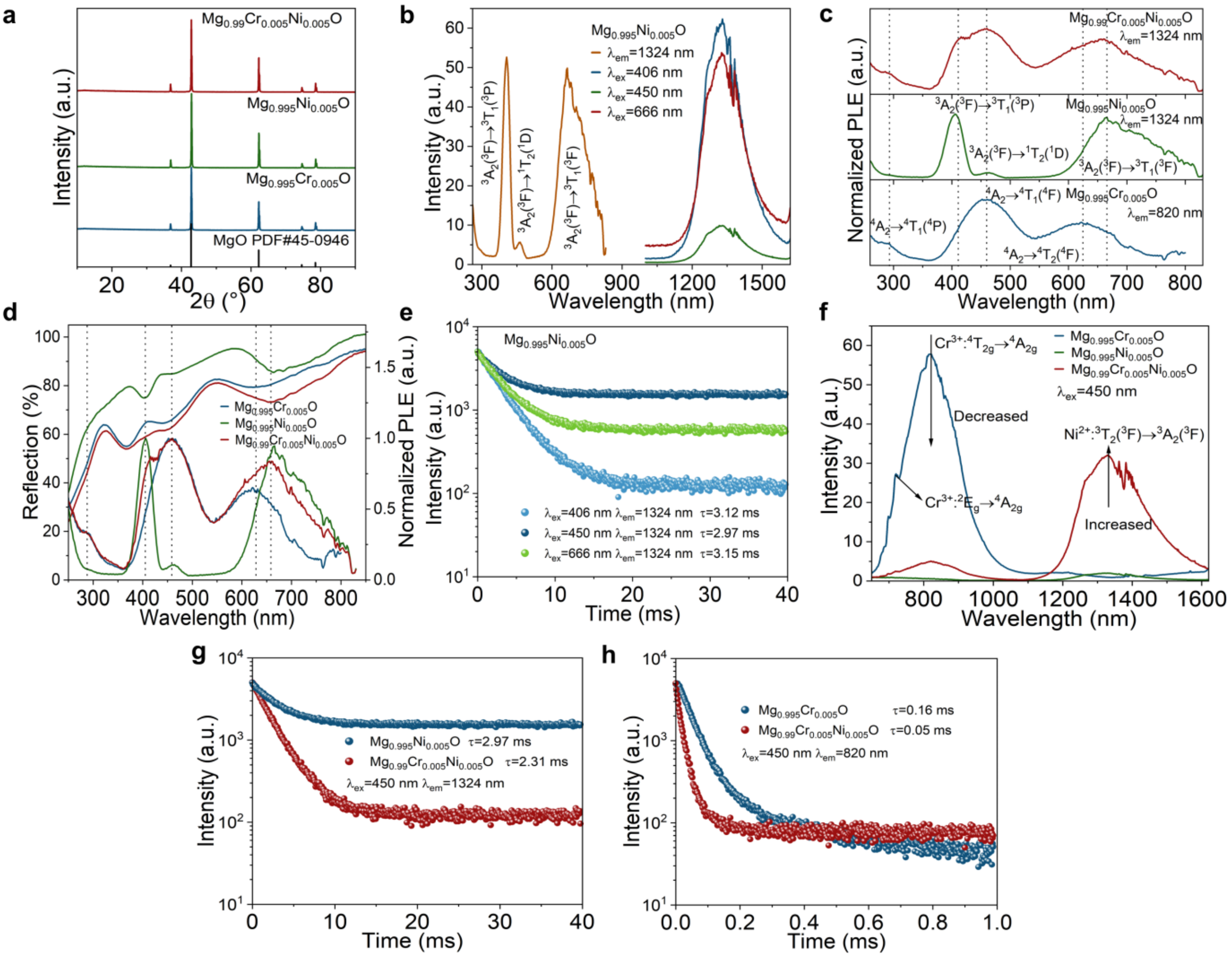

Figure S25. The efficient energy transfer between IVCT state and Ni^{2+} ion for $Mg_{0.99}Cr_{0.005}Ni_{0.005}O$, $Mg_{0.995}Cr_{0.005}O$ and $Mg_{0.995}Ni_{0.005}O$. (a) XRD patterns and standard card of MgO (PDF # 45-0946). (b) PLE and PL spectra. (c) Normalized PLE spectra. (d) PLE and DR spectra. (e) Fluorescence lifetime decay curves. (f) PL spectara. (g-h) Fluorescence lifetime decay curves. Ni^{2+} ions belong to the *[Ar] 3d2* electronic configuration, which is a good broadband NIR-II luminescent ion. Notably, the $^3A_2{\rightarrow}^3T_1$ and $^3A_2{\rightarrow}^3T_2$ absorption transitions of Ni^{2+} ions are in perfect resonance with the emission of Cr^{3+} ions, and the formation of IVCT state for Cr^{3+} clusters is conducive promoting the ET efficiency[12]. Owing to the effective energy transfer from Cr^{3+} to Ni^{2+} ion, the Cr^{3+} emission exhibits a remarkable decline in the presence of Ni^{2+}, leading to an effectively blue-light-excitable Ni^{2+}-activated NIR-II phosphor[13]. Meanwhile, the NIR-II S-ML of Ni^{2+} ion has also improved significantly after high-concentration Cr^{3+} doping.

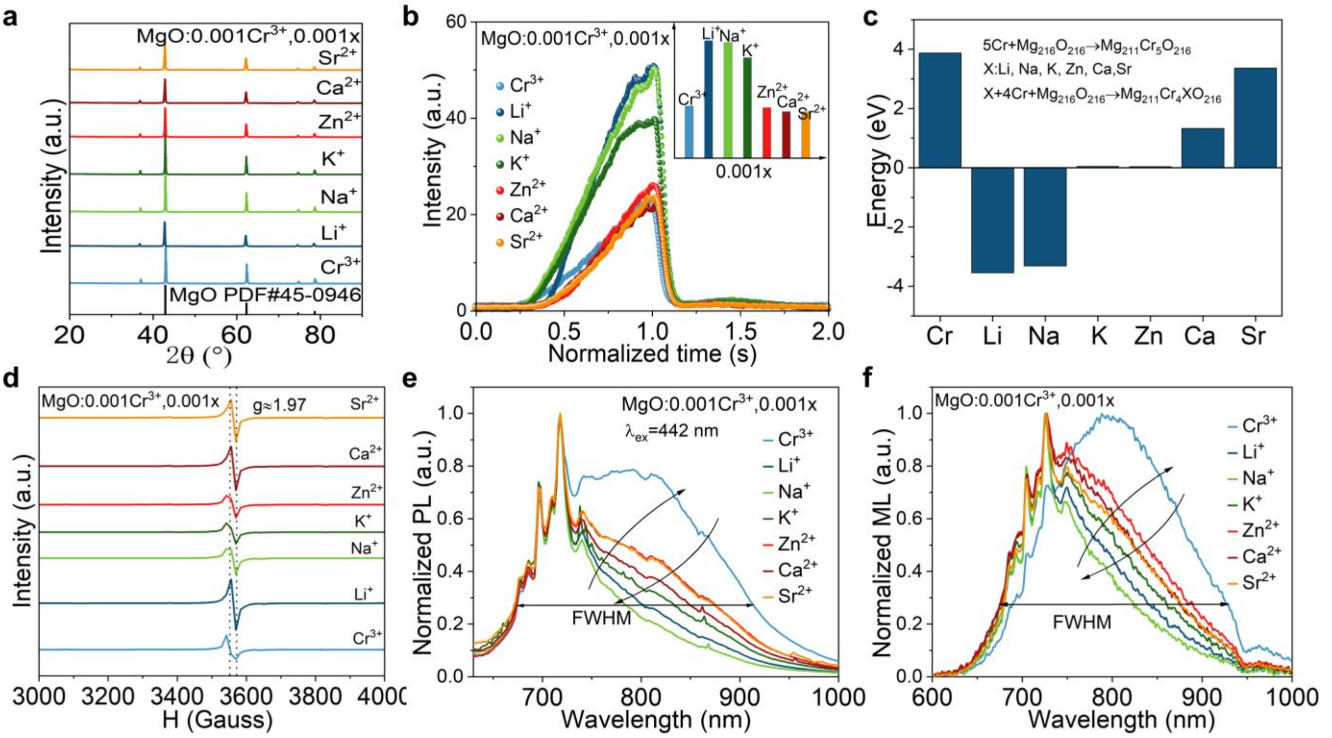

Figure S26. Lattice engineering regulates the relative content of Cr^{3+} ion pairs for MgO: $0.001Cr^{3+}$, 0.001x (x = Li^+, Na^+, K^+, Zn^{2+}, Ca^{2+}, and Sr^{2+}. (a) XRD patterns and standard card of MgO (PDF # 45-0946). (b) S-ML intensity versus test time curves under 2000 N loading. (c) Different substitution models are used to further explore the effect of external cations doping on Cr^{3+} ion pairs. It is assumed that five Cr^{3+} ions are connected to represent Cr^{3+} clusters as the initial model, other models were improved on this basis, where one cation replaced one Cr^{3+} ion to form two Cr^{3+} ion pairs. The relative magnitude of the formation energy is expressed as the degree of difficulty of Cr^{3+} ion pairs formation. (d) EPR curves. (e) Normalized PL spectra. (f) Normalized S-ML spectra.

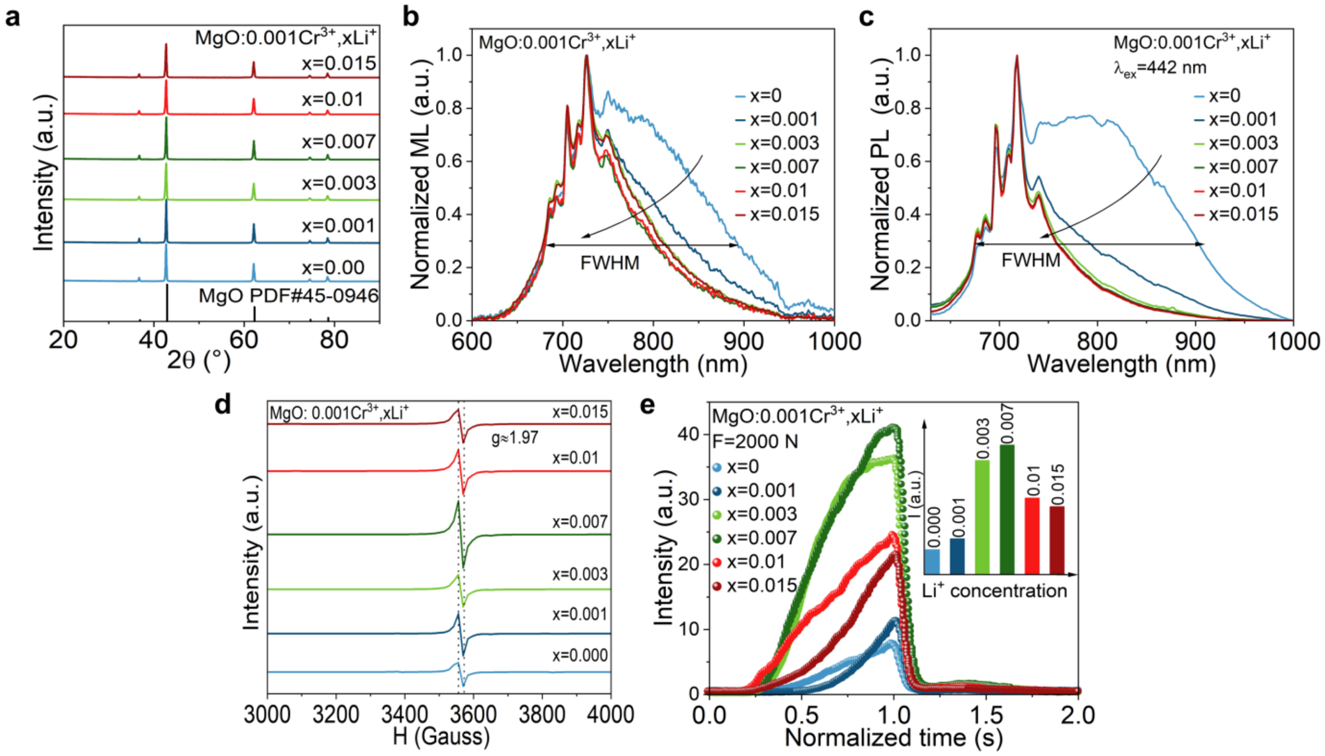

Figure S27. Li-doped samples with different concentrations were synthesized to further verify the effect Cr^{3+} ion pairs on the S-ML, including MgO: $0.001Cr^{3+}$, xLi^{+} (x = 0, 0.001, 0.003, 0.007, 0.01, and 0.015). (a) XRD patterns and standard card of MgO (PDF # 45-0946). (b) Normalized S-ML spectra. (c) Normalized PL spectra. (d) EPR curves. (e) S-ML intensity versus test time curves under 2000 N loading.

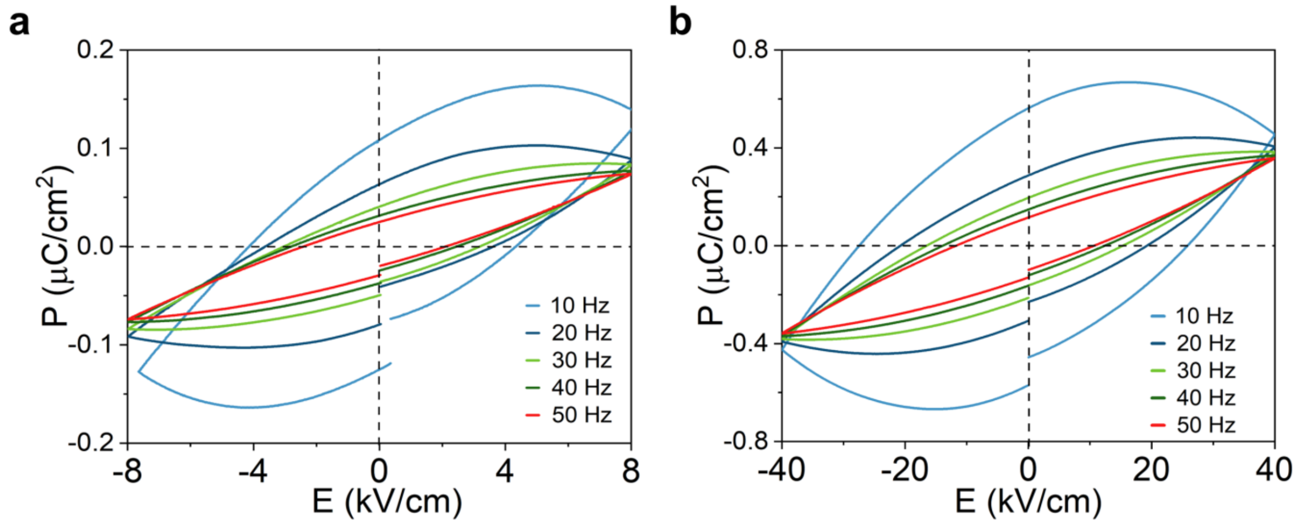

Figure S28. Room temperature P-E loop curves as a function of Hz (10, 20, 30, 40 and 50), measured at E = 8 and 40 kV/mm, respectively. The polarization hysteresis loops (*P-E*) of MgO: Cr^{3+} have relatively weak ferroelectric with slender *P-E* loops under the condition of *E* = 6 kV/mm, which demonstrates that the electrical response can be stimulated in MgO: Cr^{3+}.

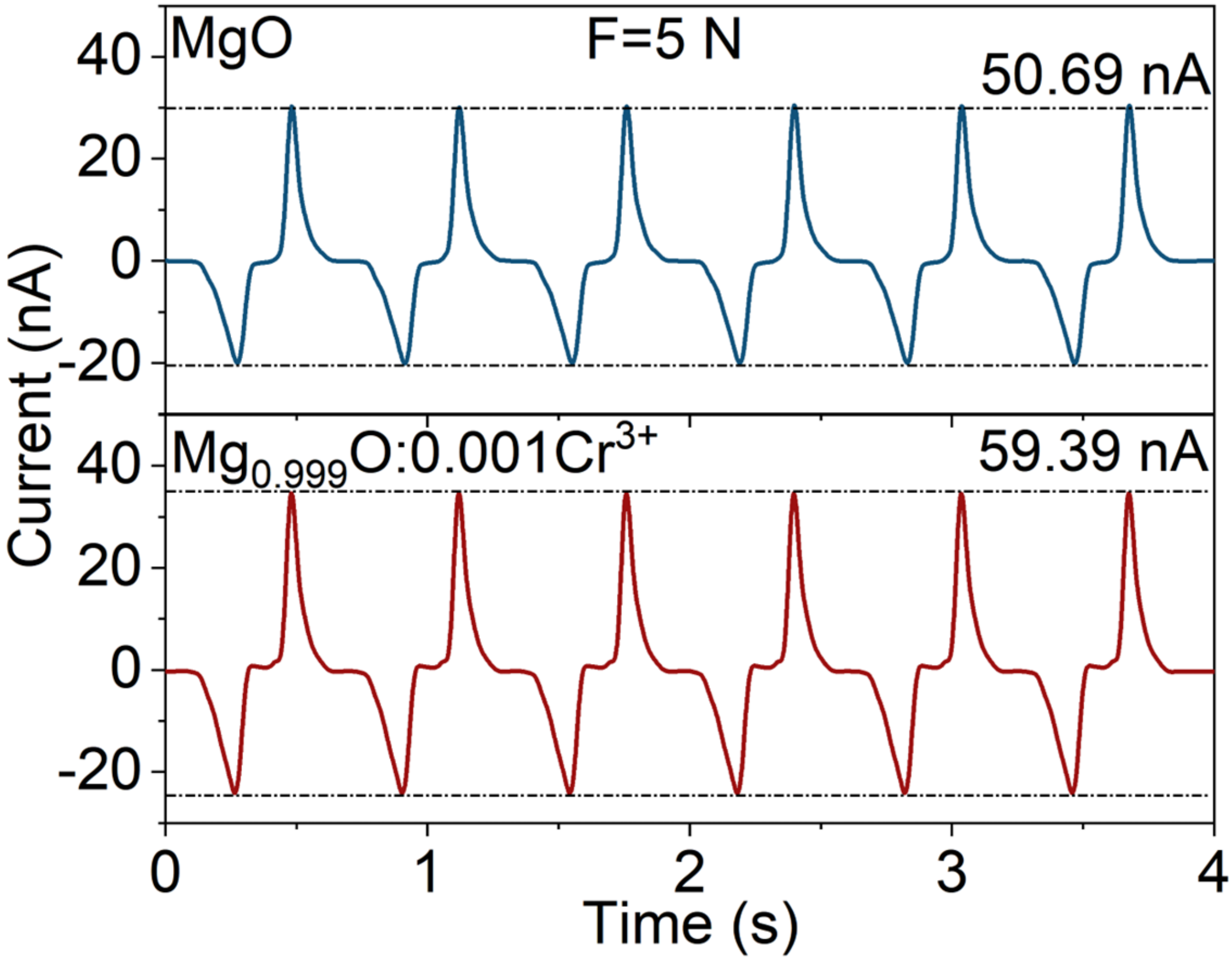

Figure S29. The stable current output for $Mg_{0.999}O$: $0.001Cr^{3+}$ and MgO under 5 N loading.

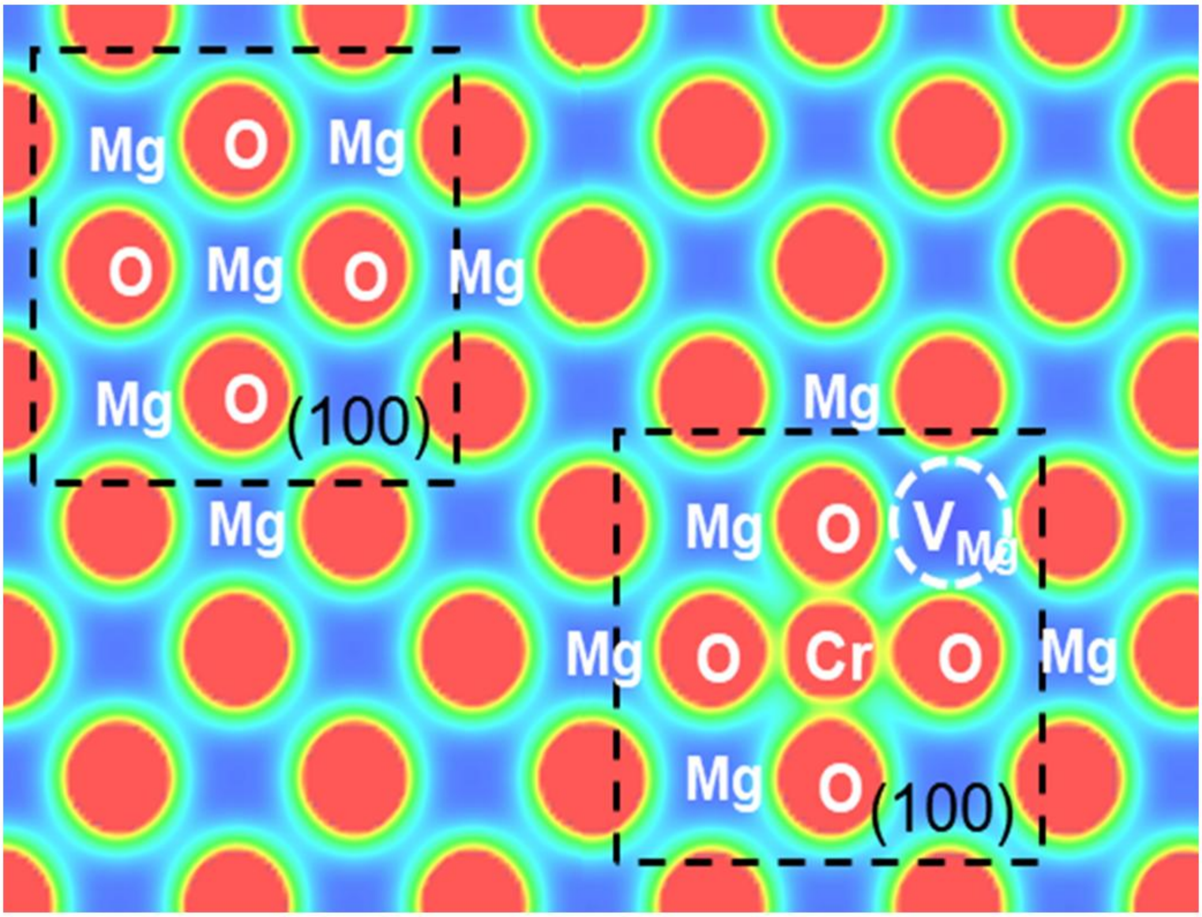

Figure S30. Two-dimensional slice of the charge distribution in MgO: Cr^{3+}.

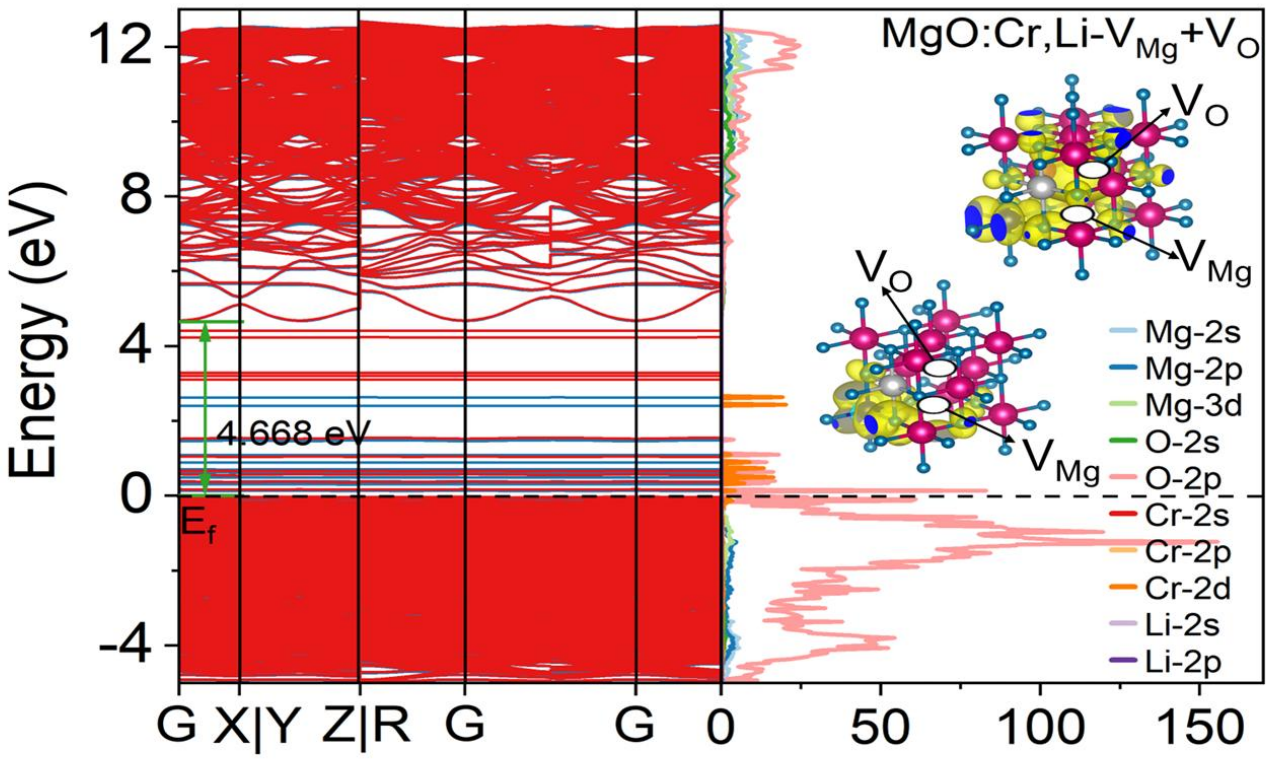

Figure S31. Band structure and electronic densities of state and the Fermi energy level for MgO: Li^{+}, Cr^{3+} with V_{Mg} and Vo.

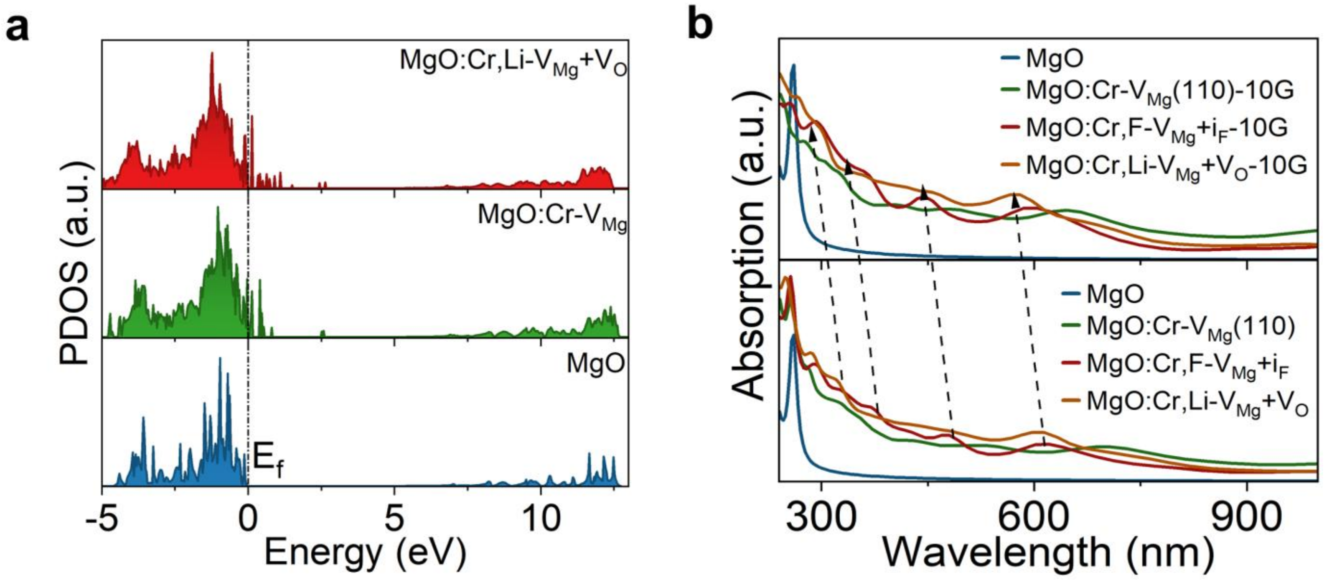

Figure S32. (a) The PDOS of O-2p of different model. (b) The absorption spectra comparisons for different model under external stress of 10 GPa.

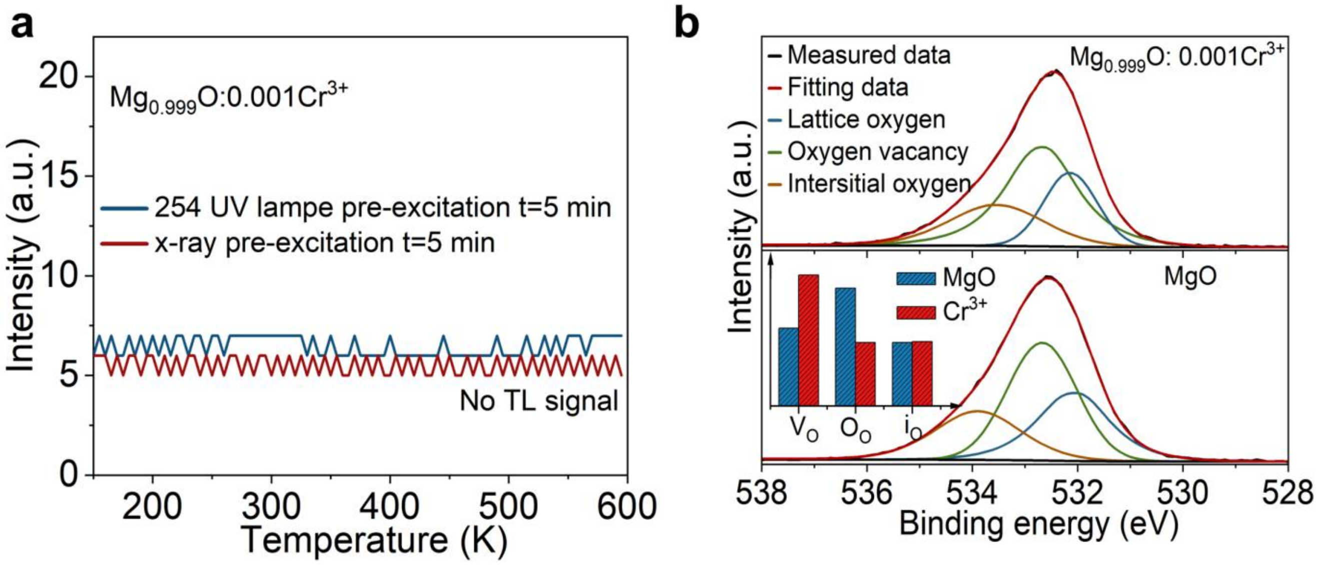

Figure S33. (a) TL curves of $Mg_{0.999}O$: $0.001Cr^{3+}$ under 254 UV lamp or X-ray pre-excitation 5 min. (b) High-revolution XPS O 1s curves of $Mg_{0.999}O$: $0.001Cr^{3+}$ and MgO and comparison of oxygen content in different states (inset).

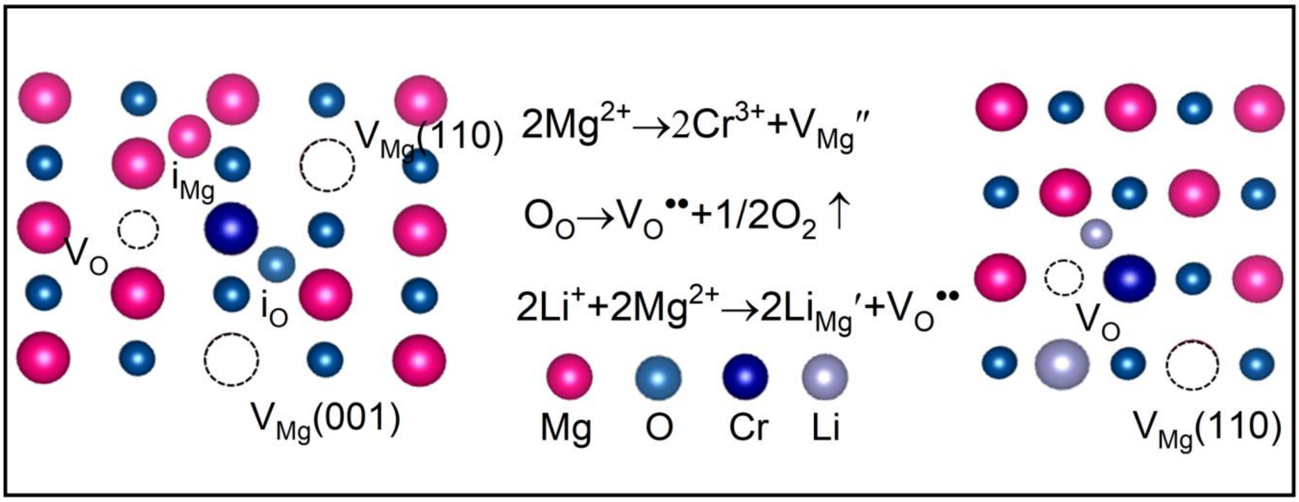

Figure S34. The defect reaction formula and corresponding various defect position after Cr^{3+} doping.

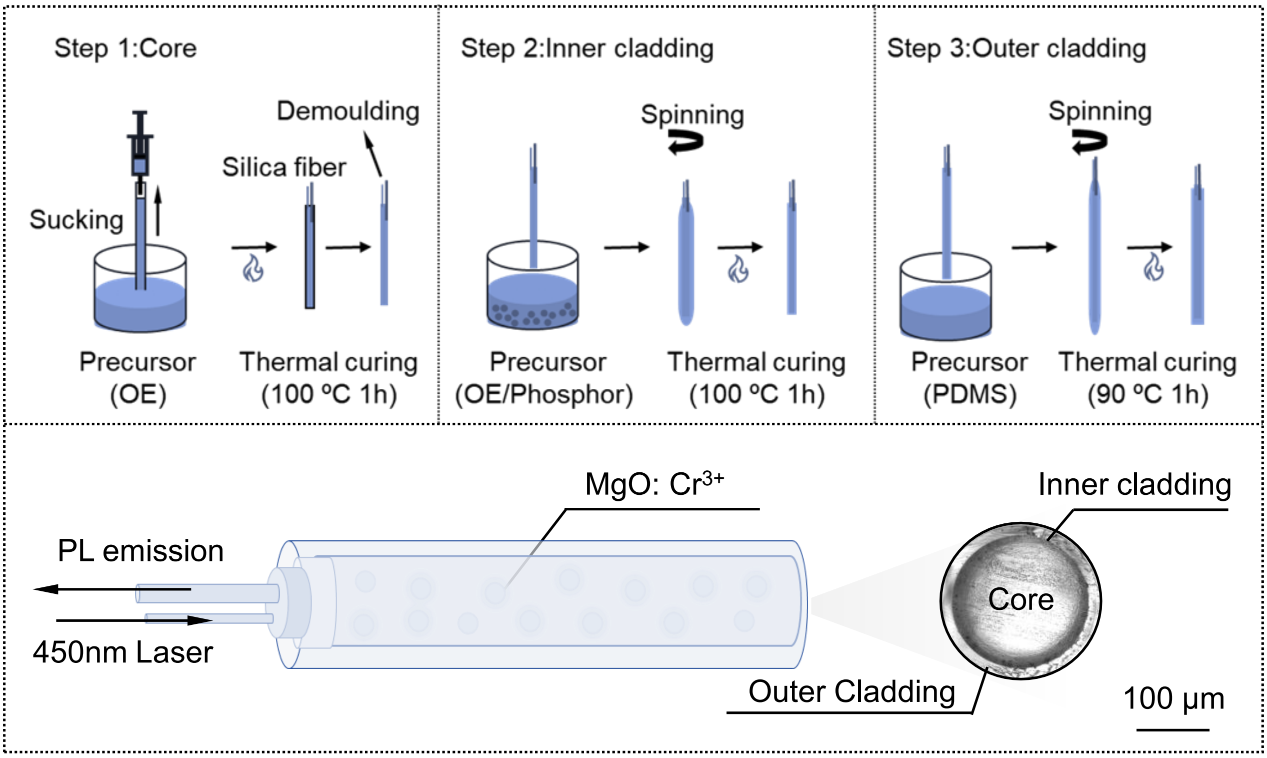

Figure S35. Preparation steps of the flexible optical fiber sensor. Inset: optical fiber cross section.

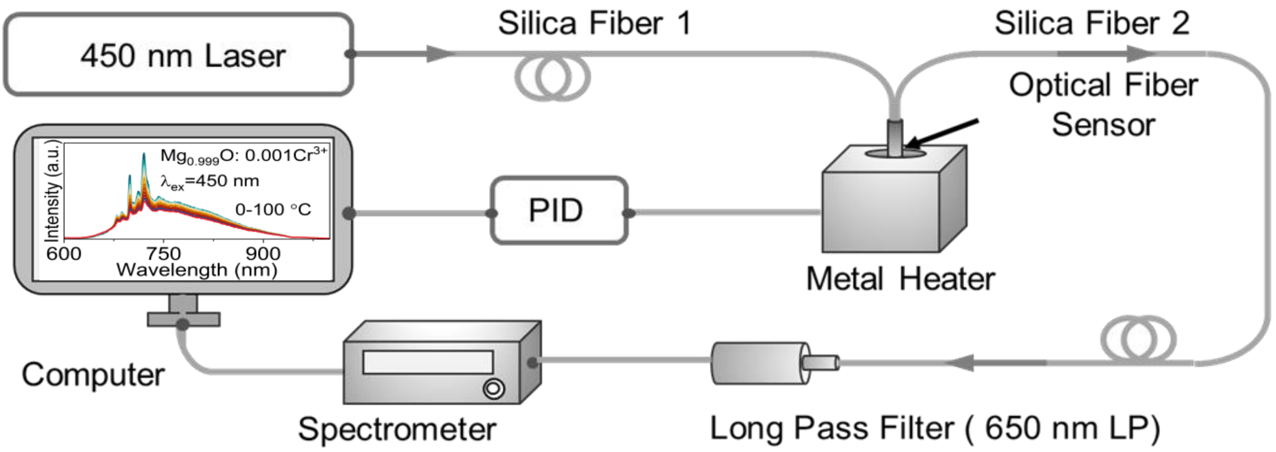

Figure S36. Flexible optical fiber sensor test platform based on multiple Cr^{3+} ion coordination state emission.

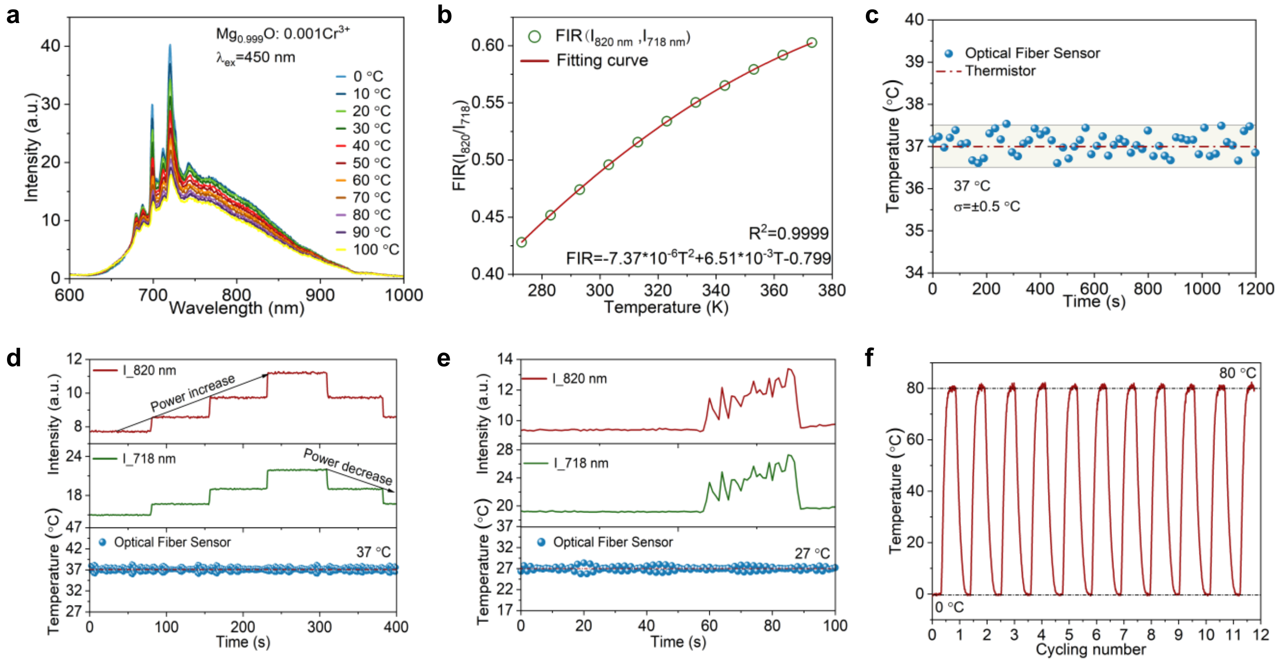

Figure S37. (a) PL spectra of optical fiber sensor ($Mg_{0.999}O: 0.001Cr^{3+}$) from 0 to 100 °C. (b) Emission intensity ratios of I_{718}/I_{820} versus the temperature based on the integrated emission intensity from 713 - 723 nm and 815 - 825 nm, respectively. (c) Fluctuations of the optical fiber sensor output over time at the constant temperature of 37 °C. The anti-disturbance performance of optical fiber sensor test: (d) Laser pump power buffeting and steep drops and (e) Shaking and swing. (f) The continue temperature cycling test of the optical fiber sensor between 0-80 °C.

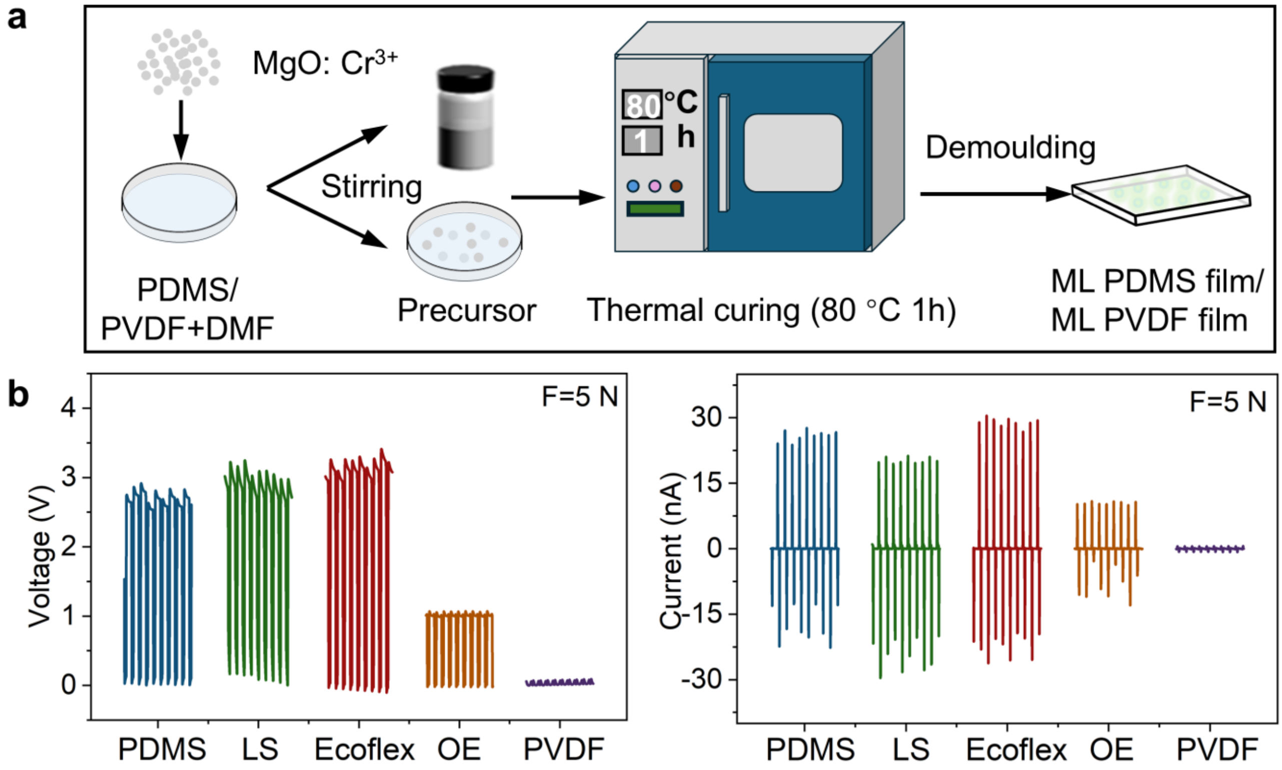

Figure S38. (a) Preparation of sandwiched sensing unit of MgO: Cr^{3+}/PDMS composite film and flexible pressure sensor. (b) The stable current and voltage output for MgO: Cr^{3+} composite with different flexible substrates under 5 N pressure. Based on the results of current and voltage data, the MgO: Cr^{3+}/PDMS film with better flexible biocompatibility is selected as the mechano-sensitive layer of the sensor after comprehensive consideration.

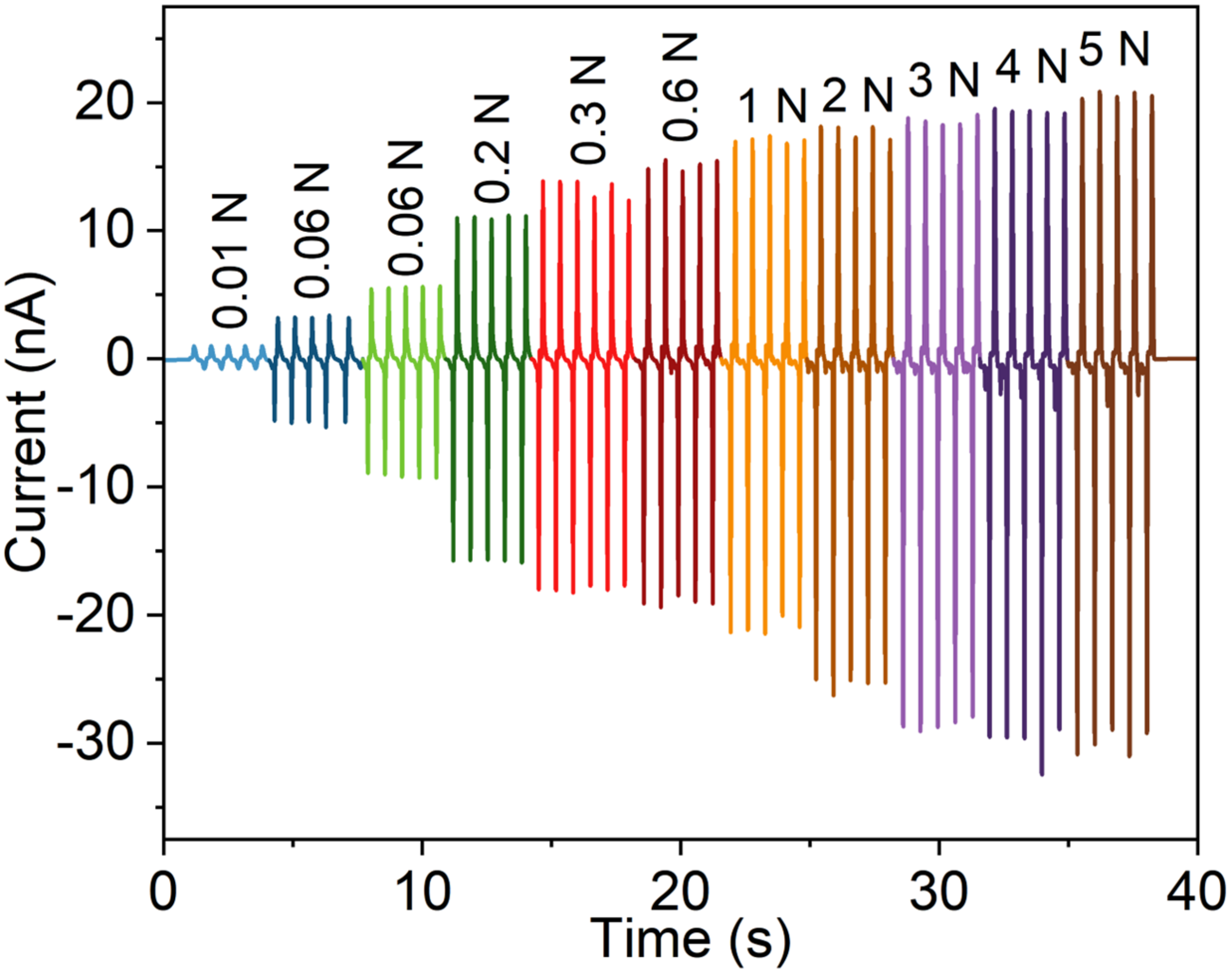

Figure S39. The stable current output for MgO: Cr^{3+} under different force from 0.01 to 5 N.

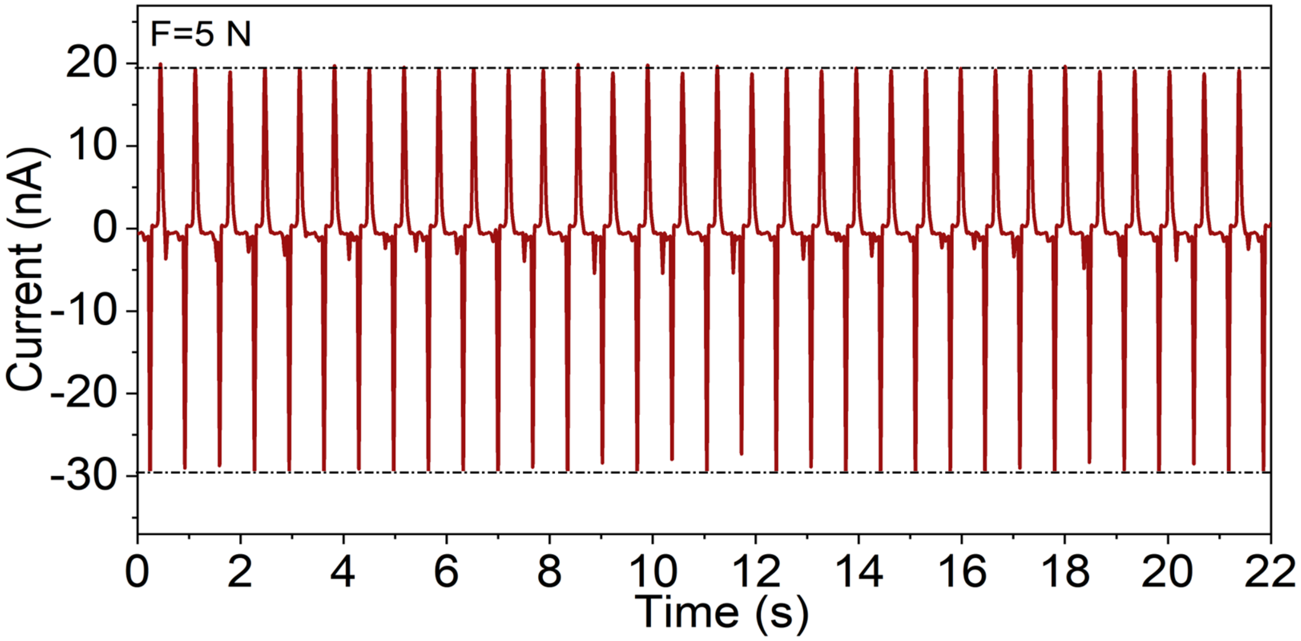

Figure S40. Cyclic test of the biomimetic sensor under continuous mechanical stimuli (F = 5 N).

Equation S8 and S9: A comprehensive investigation was conducted to elucidate the energy level configuration of Cr^{3+} pairs. When two paramagnetic transition metal ions are near each other (≤ 5 Å), they exhibit a magnetic interaction that can be described by the Heisenberg Hamiltonian H_{AB} in Equation S8, where *A* and *B* represent two transition metal ions[14].

$$H_{AB} = -2J(S_A S_B) \tag{8}$$

Where S_A and S_B represent the spin quantum numbers of transition metal ions *A* and *B*. *J* represent the exchange coupling parameter. The energy level *E(S)* of the ion pair depends on H_{AB} and is expressed by the Equation S9:

$$E(S) = -J[S(S+1) - S_A(S_A+1) - S_B(S_B+1)] \tag{9}$$

Where *S* is the total spin quantum number of the Cr^{3+} pairs. The energy is determined by the total spin *S* and the exchange coupling parameter *J*. The possible values of *S* range from the sum to the difference of S_A and S_B, with each *S* value corresponding to a unique energy level. The sign of the exchange coupling constant J in the interaction term (where $J > 0$ indicates ferromagnetic and $J < 0$ indicates antiferromagnetic coupling) depends on the type of magnetic interaction between the Cr^{3+} ions. For single Cr^{3+} ions ($3d^3$ configuration), the ground state is $^4A_2$ (S = 3/2), and the excited state is $^2E/^4T_{2g}$ (S = 1/2). When Cr^{3+} and neighboring Cr^{3+} ions exchange to form Cr^{3+} pairs, its ground state $S_A = S_B = 5/2$, so S has 5 components (S = 3, 2, 1, 0). If one of the Cr^{3+} ions is in the lowest excited state 2E and the other remains in the ground state $^4A_2$, the total spin values for the pair can be S = 2 and 1. For Cr^{3+} pairs, adopting an antiferromagnetic interaction model for both the ground state ($^4A_2 \rightarrow {}^4A_2$) and the excited state ($^4A_2 \rightarrow {}^2E/^4T_{2g}$) would yield spin alignment configurations whose splitting pattern aligns with the schematic representation[15]. S-ML and PL emissions from Cr^{3+} pairs can then be attributed to transitions from the lowest excited spin state to various spin components of the ground state. Cr^{3+} pairs are spin-allowed transitions of $\Delta S = 0$. So, the magnetic interaction of the Cr^{3+} pairs can change the spin configuration of electrons, break the d-d transition spin prohibition-selective law of isolated Cr^{3+} ion, and then leading to higher probabilities and contributing to broadband emission[16]. Notably, the Cr^{3+} pairs interaction and resulting emission properties in Cr^{3+}-doped systems are controlled by three interdependent factors: (i) the magnetic coupling between the ions; (ii) the spacing of Cr^{3+} ions within the lattice, and (iii) the symmetry of Cr^{3+} ion local

coordination environments, resulting in a dual emission profile comprising narrow R-line from isolated Cr^{3+} centers and broadened emissions originating from exchange-coupled Cr^{3+}-pairs[10].

Table S1. Different atomic site occupancy and select interatomic distances of $Mg_{0.999}O$: $0.001Cr^{3+}$

Space group	cubic	Symmetry	Fm-3m (225)			
Cell parameters	a = b = c = 4.2084 Å, α = β = γ = 90º, V=74.5634 $Å^3$					
Reliability factors	R_p=8.67 %, R_{wp} = 12.3 %, χ^2=5.11					
Atom	x	y	z	Occ. (%)	Bond type	Bond length (Å)
Mg	0.000	0.000	0.000	99.039	Mg-O	2.1042
O	0.500	0.500	0.500	100		
Cr	0.000	0.000	0.000	0.0961		

Table S2. The calculated defect formation energies, average Mg-O bond lengths, and distortion indexes after Cr^{3+} doping. The average Mg-O bond length is 2.10354 Å in undoped MgO

Model	Energy (eV) V_{Mg} (001)	Energy (eV) V_{Mg} (110)	After doping Average Mg-O bond length (Å)	After doping distortion index
I	0.72102	1.00602	2.1091	0.0071
II	1.16352	0.95442	2.0984	0.0029
III	0.72102	1.00602	2.1091	0.0071
IV	1.16352	0.90512	2.0984	0.0029
V	1.16352	0.95442	2.1187	0.0056
VI	0.72102	1.00602	2.1091	0.0071

The distortion indexes were calculated based on Equation S1.

Table S3. EXAFS data fitting results of $Mg_{0.995}O:0.005Cr^{3+}$

Sample	Path	CN^a	R(Å)^b	σ^2 (Å^2)^c	ΔE_0(eV)^d	R factor
Cr K-edge ($S_0{}^2$=0.763)						
Cr foil	Cr-Cr	8.0*	2.491	0.0059	4.3 ±1.5	0.0019
	Cr-Cr	6.0*	2.869	0.0047		
Cr_2O_3	Cr-O	6.2±0.5	1.992	0.0039	2.5 ± 2.6	0.0208
	Cr-O-Cr	2.7±0.3	2.899	0.0033	7.7 ± 3.8	
	Cr-O-Cr	2.9±0.7	3.438	0.0040	3.2 ± 6.8	
	Cr-O-Cr	4.4±1.0	3.696			
CrO_2	Cr-O	6.2±0.5	1.911	0.0041	2.7 ± 2.6	0.0193
	Cr-O-Cr	1.9±0.3	2.930	0.0033	7.1 ± 1.4	
	Cr-O-Cr	6.0±0.4	3.452			
sample	Cr-O	4.7±0.4	1.985	0.0057	5.4 ± 2.5	0.0181
	Cr-O-Cr	2.2±0.5	2.954	0.0097	12.8 ± 6.5	

[a]*CN*, coordination number; [b]*R*, the distance between absorber and backscatter atoms; [c]σ^2, the Debye Waller factor value; [d]ΔE_0, inner potential correction to account for the difference in the inner potential between the sample and the reference compound; *R* factor indicates the goodness of the fit. $S0^2$ was fixed to 0.763, according to the experimental EXAFS fit of Cr foil by fixing *CN* as the known crystallographic value. This value was fixed during EXAFS fitting, based on the known structure of Cr. Fitting conditions: *k* range：3.0 - 11.5; *R* range: 1.0-3.0; fitting space: R space; *k*-weight = 3. A reasonable range of EXAFS fitting parameters: $0.800 < S_0{}^2 < 1.000$; $CN > 0$; $\sigma^2 > 0$ Å^2; $|\Delta E_0| < 15$ eV; R factor < 0.02.

Table S4. Luminescence lifetime of of $Mg_{1-x}O$: xCr^{3+} under λ_{ex} = 442 nm and λ_{em} = 718 nm

x	τ (ms)	R^2
0.000005	2.2933 ± 0.0046	0.999
0.00001	2.2908 ± 0.0073	0.999
0.00005	2.1946 ± 0.0059	0.999
0.0001	2.0856 ± 0.0064	0.999
0.0005	1.9334 ± 0.0069	0.999
0.001	1.4659 ± 0.0120	0.997
0.003	0.8973 ± 0.0082	0.997
0.007	0.5170 ± 0.0039	0.999
0.01	0.4462 ± 0.0046	0.998
0.015	0.4243 ± 0.0064	0.997

Table S5. Luminescence lifetime of of $Mg_{1-x}O$: xCr^{3+} under λ_{ex} = 442 nm and λ_{em} = 820 nm

**x**	**τ (ms)**	**R^2**
0.000005	-	-
0.00001	-	-
0.00005	1.8388 ± 0.0687	0.994
0.0001	1.5841 ± 0.0687	0.996
0.0005	0.8215 ± 0.0327	0.997
0.001	0.4532 ± 0.0576	0.997
0.003	0.3449 ± 0.0880	0.997
0.007	0.2582 ± 0.0885	0.998
0.01	0.1526 ± 0.0798	0.999
0.015	0.1275 ± 0.0281	0.999

Table S6. Cr^{3+}-activated S-ML phosphors (Year: 2021-2025)

host	Crystal structure	Space group	Cr^{3+} pair	S-ML	Reference
Ga_2O_3	Monoclinic	C/2m	Yes	Yes	[17]
$MgGa_2O_4$	Cubic	Fd-3m	Yes	Yes	[18]
$ZnGa_2O_4$	Cubic	Fd-3m	Yes	Yes	[18]
$LaAlO_3$	Cubic	R-3c	Yes	Yes	[19]
$LiGa_5O_8$	Cubic	$P4_332$	Yes	Yes	[20]
Al_2O_3	Hexagonal	$P6_3/mmc$	Yes	Yes	[21]
$NaAl_{11}O_{17}$	Hexagonal	$P6_3/mmc$	Yes	Yes	[10]
$NaGa_{11}O_{17}$	Hexagonal	$P6_3/mmc$	Yes	Yes	[10]
$SrAl_{12}O_{19}$	Hexagonal	$P6_3/mmc$	Yes	Yes	[22]
$LaMgGa_{11}O_{19}$	Hexagonal	$P6_3/mmc$	Yes	Yes	[23]
$Zn_3Ga_2GeO_8$	Cubic	Fd-3m	No	No	[22]
$Y_3Al_5O_{12}$	Cubic	Ia-3d	No	No	[24]
Sr_2GaSbO_6	Tetragonal	I4/m	Yes	Yes	[25]
Sr_2SrSbO_6	Monoclinic	$P2_1/n$	Yes	Yes	[25]
Ba_2ScSbO_6	Cubic	Fm-3m	Yes	Yes	[25]
$Lu_3Ga_5O_{12}$	Cubic	Ia-3d	No	No	[26]
$Y_3Ga_3MgSiO_{12}$	Cubic	Ia-3d	No	No	[27]
MgO	Cubic	P1	Yes	Yes	This work

One can use the relative intensity of the EPR curve at g = 1.97 to evaluate the relative content of Cr^{3+} pair and of Cr^{3+} cluster within the S-ML materials.

References

[1] S. I. Zabinsky, J. J. Rehr, A. Ankudinov, R. C. Albers, M. J. Eller, *Physical Review B* **1995**, 52, 2995.

[2] G. Kresse, J. Furthmüller, *Phys. Rev. B* **1996**, 54, 11169.

[3] C. Freysoldt, B. Grabowski, T. Hickel, J. Neugebauer, G. Kresse, A. Janotti, C. G. Van de Walle, *Rev. Mod. Phys.* **2014**, 86, 253.

[4] Q. Bai, S. Zhao, L. Guan, Z. Wang, P. Li, Z. Xu, *Cryst. Growth Des.* **2018**, 18, 3178.

[5] F. Zhao, Z. Song, Q. Liu, *Laser Photonics Rev.* **2022**, 16, 2200380.

[6] Y. Zhou, X. Li, T. Seto, Y. Wang, *ACS Sustainable Chem. Eng.* **2021**, 9, 3145.

[7] X. Ding, G. Zhu, W. Geng, Q. Wang, Y. Wang, *Inorg. Chem.* **2016**, 55, 154.

[8] L. Van Uitert, *J. Electrochem. Soc.* **1967**, 114, 1048.

[9] F. Xiao, C. Xie, R. Yi, H. Yuan, Q. Zhou, *Optical Materials* **2022**, 125, 112131.

[10] Q. Pang, Y. Wang, L. Yan, G. Zhu, S. Xu, J. Zhang, X. Zhang, Y. Cao, B. Chen, *Laser Photonics Rev.* **2023**, 18, 2301039.

[11] H. Liu, F. Zhao, H. Cai, Z. Song, Q. Liu, *J. Mater. Chem. C* **2022**, 10, 9232.

[12] B.-M. Liu, X.-X. Guo, L.-Y. Cao, L. Huang, R. Zou, Z. Zhou, J. Wang, *Chem. Eng. J.* **2023**, 452.

[13] a)S. Gu, B. Liu, S. Si, J. Wang, *J. Mater. Chem. C* **2023**, 11, 9014; b)S. Liu, Y. Guo, M. Zhao, J. Du, Z. Song, X. Zhang, F. Wang, Q. Liu, *Laser Photonics Rev.* **2024**, 18, 2400475.

[14] M. A. d. B. A. P. Vink, S. Roke, P. S. Peijzel and A. Meijerink, *J. Electrochem. Soc.* **2001**, 148, E313.

[15] X. Zhang, L. Zhou, H. You, *Adv. Mater.* **2025**, 37, 2419897.

[16] E. Song, S. Ye, T. Liu, P. Du, R. Si, X. Jing, S. Ding, M. Peng, Q. Zhang, L. Wondraczek, *Adv. Sci.* **2015**, 2, 1500089.

[17] H. Suo, Y. Wang, X. Zhang, W. Zheng, Y. Guo, L. Li, P. Li, Y. Yang, Z. Wang, F. Wang, *Matter* **2023**, 6, 2935.

[18] P. Shao, D. Chen, Z. Lun, Y. Wu, Z. Chen, Y. Xiao, P. Xiong, S. Wang, B. Viana, W. B. Im, Z. Yang, *Small* **2024**, 20, 2402352.

[19] P. Shao, P. Xiong, Y. Xiao, Z. Chen, D. Chen, Z. Yang, *Adv. Powder Mater.* **2024**, 3, 100165.

[20] P. Xiong, B. Huang, D. Peng, B. Viana, M. Peng, Z. Ma, *Adv. Funct. Mater.* **2021**, 31, 2010685.

[21] H. Zhu, Y. Li, Y. Xi, C. Xin, C. Zhou, Z. Yang, L. Ruan, Y. Li, Y. Peng, M. S. Molokeev, A. Zolotov, J. Wang, Z. Zhou, M. Xia, *Laser Photonics Rev.* **2024**, 19, 2401089.

[22] S. Liu, Y. Zheng, D. Peng, J. Zhao, Z. Song, Q. Liu, *Adv. Funct. Mater.* **2022**, 33, 2209275.

[23] S. Liu, J. Du, Z. Song, C. Ma, Q. Liu, *Light: Sci. Appl.* **2023**, 12, 181.

[24] Z. Liu, X. Yu, Q. Peng, X. Zhu, J. Xiao, J. Xu, S. Jiang, J. Qiu, X. Xu, *Adv. Funct. Mater.* **2023**, 33, 2214497.

[25] C. Dou, T. Liang, M. Zhao, Z. Song, L. Ning, D. Peng, Q. Liu, *Adv. Funct. Mater.* **2024**, 35, 2419716.

[26] S. Wu, B. Xiao, Y. Xiao, P. Shao, Y. Wang, P. Xiong, *Nano Energy* **2023**, 116, 108811.

[27] S. Wu, G. Zhou, Y. Wu, P. Xiong, B. Xiao, Z. Zhou, Y. Xiao, P. Shao, S. Wang, Z. Shao, Y. Wang, F. Wang, *Adv. Mater.* **2024**, 36, 2408508.
